# Cognition in cerebellar disorders: What’s in the profile? A systematic review and meta-analysis

**DOI:** 10.1007/s00415-025-12967-8

**Published:** 2025-03-06

**Authors:** Stacha F. I. Reumers, Fleur L. P. Bongaerts, Frank-Erik de Leeuw, Bart P. C. van de Warrenburg, Dennis J. L. G. Schutter, Roy P. C. Kessels

**Affiliations:** 1 Department of Neurology, Radboud University Medical Centre, Donders Institute for Brain, Cognition, and Behaviour, Nijmegen, The Netherlands; 2 Helmholtz Institute, Department of Experimental Psychology, Utrecht University, Utrecht, The Netherlands; 3 Donders Institute for Brain, Cognition and Behaviour, DCC–Neuropsychology & Rehabilitation Psychology, Radboud University, Nijmegen, The Netherlands; 4 Radboud University Medical Centre, Radboudumc Alzheimer Centre, Nijmegen, The Netherlands; 5 Vincent Van Gogh Institute for Psychiatry, Venray, The Netherlands

**Keywords:** Cerebellar cognitive affective syndrome, Cognitive dysfunction, Neuropsychological assessment, Meta-analysis

## Abstract

**Objective:**

This systematic review and meta-analysis aim to examine the profile and extent of cognitive deficits in patients with cerebellar disorders, and to provide a complete overview of the cognitive domains that might be affected in the Cerebellar Cognitive Affective Syndrome (CCAS).

**Methods:**

MEDLINE, Embase, PsycINFO, and Web of Science were systematically searched to 17-07-2024. Studies were considered if the participants were adult patients with a clinical diagnosis of cerebellar disorder and were neuropsychological assessed. Outcomes were grouped into the domains of processing speed, language, social cognition, executive function, visuospatial skills, episodic memory, verbal intelligence, attention, and working memory. All aetiologies were included for first evaluation and patients were assigned to one of two groups (focal vs. degenerative) for secondary evaluation. Random-effects models were employed for the meta-analyses.

**Results:**

129 studies with a total of 3140 patients with cerebellar disorders were included. Patients performed significantly worse compared to control/standardized data in all domains. Deficits were most pronounced in processing speed, ES [95% CI] = − 0.83 [− 1.04, − 0.63], language, ES [95% CI] = − 0.81 [− 0.94, − 0.67], and social cognition, ES [95% CI] = − 0.81 [− 1.19, − 0.42]. Cognitive impairment varied between patients with focal cerebellar lesions and degenerative cerebellar disorders, but was overall worse in the degenerative group.

**Discussion:**

Cerebellar disorders can impact many cognitive domains, extending beyond executive functioning, visuospatial skills, and language. These outcomes contribute to a broader understanding of the cerebellum's role in cognition and sheds light on the cognitive deficits associated with cerebellar disorders.

**Supplementary Information:**

The online version contains supplementary material available at 10.1007/s00415-025-12967-8.

## Introduction

Cognitive deficits related to cerebellar pathologies were first described by Schmahmann and Sherman in 1998 as the Cerebellar Cognitive Affective Syndrome (CCAS). It is defined by impairments in four key characteristic cognitive and behavioral domains [1, 2]. First, executive functioning, including set-shifting, working memory, and abstract reasoning. Second, language, such as agrammatism and dysprosody. Third, spatial cognition, including visual–spatial memory and organization. Finally, deficits in affect and behavioral changes, such as disinhibited behavior and blunting of affect [3]. These symptoms have a profound impact on the daily life of patients [4–6]. Cognitive dysfunction in disorders of the cerebellum has been increasingly described in the literature, but a comprehensive overview of the cognitive profile is to date lacking. The cerebellum with its connections to the supratentorial association areas, basal ganglia, midbrain, and limbic system is nowadays recognized as a contributor to cognitive processes [7]. Disruption of the cerebellar circuits may result in cognitive impairments, but the underlying mechanisms are still largely unknown [8, 9].

In the last 25 years, CCAS has received increasingly more scientific and clinical attention. CCAS has been studied in various cerebellar disorders, including isolated (mostly vascular) cerebellar lesions, Friedreich’s ataxia, and spinocerebellar ataxia type 3 (SCA3) [10–12]. These studies have often been small studies or case reports, assessing various cognitive domains with different neuropsychological tests, and were mostly limited to specific etiologies. These large methodological differences pose a challenge to directly compare outcomes across studies. Even among studies evaluating a single etiology, contradictory findings have been reported with regard to the domains affected. Moreover, multiple studies suggest that beyond the four key domains as initially described, difficulties with (verbal) memory, procedural learning, and sequencing are also part of the spectrum of cognitive deficits related to cerebellar pathologies [5, 13–16]. On the same note, there is increasing evidence for involvement of the social-cognitive domain in cerebellar disorders, which is not included in the original description of CCAS [17, 18]. Considering recent developments in the field of CCAS, a comprehensive review of the cognitive profile in cerebellar disorders may yield important insights into the cognitive sequelae of cerebellar disease.

The aim of this meta-analysis was to examine the extent and profile of cognitive impairments in patients with cerebellar disorders compared to healthy controls. Up-to-date knowledge about the cognitive profile is important as this may have implications for the diagnosis, management, and possible treatment of cognitive deficits. Furthermore, it could be helpful for clinicians to inform patients about the possible cognitive sequelae of cerebellar disorders, as well as guiding future research efforts. To provide a complete overview of the cognitive domains that might be affected in CCAS, etiologies with primary cerebellar pathology will be included. To gain further insight into the specific contribution of the cerebellum to cognitive deficits, data will be aggregated in patient groups with focal lesions isolated/limited to the cerebellum and degenerative cerebellar disorders. Disparities between types of cerebellar disorders are expected due to divergent brain pathology. Given the widespread atrophy and possible extracerebellar involvement in patients with degenerative disorders, more extensive cognitive impairment is anticipated in this group compared to those with focal lesions.

## Methods

The Preferred Reporting Items for Systematic Reviews and Meta-Analyses (PRISMA) guidelines were followed [19, 20]. This study was pre-registered in the international prospective register of systematic reviews (PROSPERO; CRD42023482954).

### Study selection and eligibility criteria

Studies published in peer-reviewed journals were included if the study population consisted of adults (≥ 18 years) with a clinical diagnosis of a cerebellar disorder. All etiologies with primary cerebellar pathology were included, and patients were subdivided into two groups for further exploration: (1) patients with focal isolated lesions confined to the cerebellum (surgical, ischemic, or hemorrhagic), and (2) patients with degenerative disorders (both hereditary and sporadic types) characterized by cerebellar atrophy. Patients with multiple system atrophy (MSA) were only included if this was the cerebellar subtype (MSA-C). Cognitive functioning was examined with objective, validated neuropsychological tests, which could be either assessed by paper-and-pencil or computerized. Subjective-only outcome measures (e.g., questionnaires) were excluded, as well as screeners such as the Montreal Cognitive Assessment (MoCA), Mini-Mental State Examination (MMSE), and CCAS scale. Individual test results were included if they allowed for effect sizes to be calculated. This included data for which a healthy control group was used for comparison, or normative referenced data (standardized z scores, t scores, and percentiles). When outcomes were reported for multiple timepoints, the first evaluation (indicative of the shortest disease duration) was taken. If information in the article was incomplete, the corresponding author was contacted to provide specific data.

Only empirical studies were considered with no language restrictions. To avoid duplicates, articles that did not report original data were excluded. If multiple articles reported outcomes for the same study sample, the article with the most comprehensive dataset was included to avoid overlap. Conference summaries/abstracts, reviews, and single case studies were excluded.

### Search strategy

A systematic literature search was conducted using the following electronic databases: Embase, MEDLINE, PsycINFO, and Web of Science. In addition, a manual search was conducted, by screening references from included studies and related reviews. Search terms were compiled together with an experienced librarian from the Radboud University library, and were applied to title, abstract, and keywords. Articles were identified by the search terms: ‘cerebellar disorder’, ‘cognition’, and related terms (Supplementary Information 1). The search was carried out on November 14, 2023 and updated on July 17, 2024. Two reviewers (S.R. & F.B.) reviewed the articles independently, and in case of disagreement, a consensus was reached by a third researcher (R.P.C.K.). Search results were exported to Covidence systematic review software for selection and review processes.

### Data extraction and outcomes

Extracted data included study year, sample size, study restrictions, participant demographics (i.e., country, sex, age, and education), cerebellar disorder characteristics (i.e., type, age at onset, and disease duration), lesion locations for focal lesions, and primary outcome measurements. When raw outcomes were not reported as means with standard deviations, these were estimated from the standard error of the mean (SEM, SD = SEM * √n), the 95% CI (SD = √n * (CI upper limit–CI lower limit) / 3.92), or the median and IQR using appropriate methodology [21]. Primary outcome measures were performance on neuropsychological tests, and to provide a comprehensive view of the cognitive profile, outcomes were grouped in cognitive domains according to Lezak et al. [22], and in consultation with the senior author who is a clinical neuropsychologist (R.P.C.K.). The following cognitive domains were distinguished: verbal intelligence, visuospatial skills, language, attention, executive functions, working memory, episodic memory, processing speed, and social cognition. These were, if possible, also divided in subdomains (e.g., emotion recognition and theory of mind for social cognition). An overview of the tests with associated (sub-)domains are provided in Supplementary Information 2.

### Quality assessment

Quality assessment and risk of bias of the included articles was based on critical appraisal tools and the Risk of Bias Assessment Tool for Nonrandomized Studies (RoBANS) [23, 24]. Criteria evaluated the (reporting) quality and suitability of participant selection, data collection, outcomes, and risk of bias (Supplementary Information 3).

### Analyses

Cognitive outcomes were quantified using transformation to Hedge’s g effect size to correct for small sample sizes. Effect sizes (ES) and 95% confidence intervals (95% CI) were computed for all cognitive outcome measures and then aggregated into one effect size per test, and subsequently into cognitive domains and subdomains. A random-effects meta-analysis for all cognitive domains and subdomains was performed on the entire group (all etiologies) for the primary analysis. For secondary analyses, analyses for all cognitive domains were performed on subgroups (focal group vs. degenerative group). For the three cognitive domains with large effect sizes, the effect sizes of subdomains were explored as well.

Random-effects models were employed as these account for heterogeneity between studies [25]. Statistical analyses were performed using RStudio 3.6.2 (Metafor package) [26] and the significance level was set at 0.05. The R code used for analyses is shared via Open Science Framework (https://osf.io/3a95q/). For the interpretation of outcomes, effect sizes of 0.2–0.5 were considered small, 0.5–0.8 as medium, and > 0.8 as large [27]. Results were visualized with forest plots. In addition, a sensitivity and heterogeneity analysis using I^2^ and τ^2^ statistics was performed. Statistics of I^2^ provide a measure for the proportion of observed variance reflecting real differences in effect size, whereas τ^2^ provides an estimate of the true effect size variance [28]. To assess potential publication bias, the Egger’s regression test was performed and funnel plots were inspected for asymmetry per cognitive domain. The general method of the fail-safe *N* approach (alpha level = 0.05) was used to calculate the minimum number of studies averaging null results that would have to be added to change the meta-analytic result. Small fail-safe *N* numbers indicate that the results are not robust to publication bias.

## Results

### Literature search

**Fig. 1:**
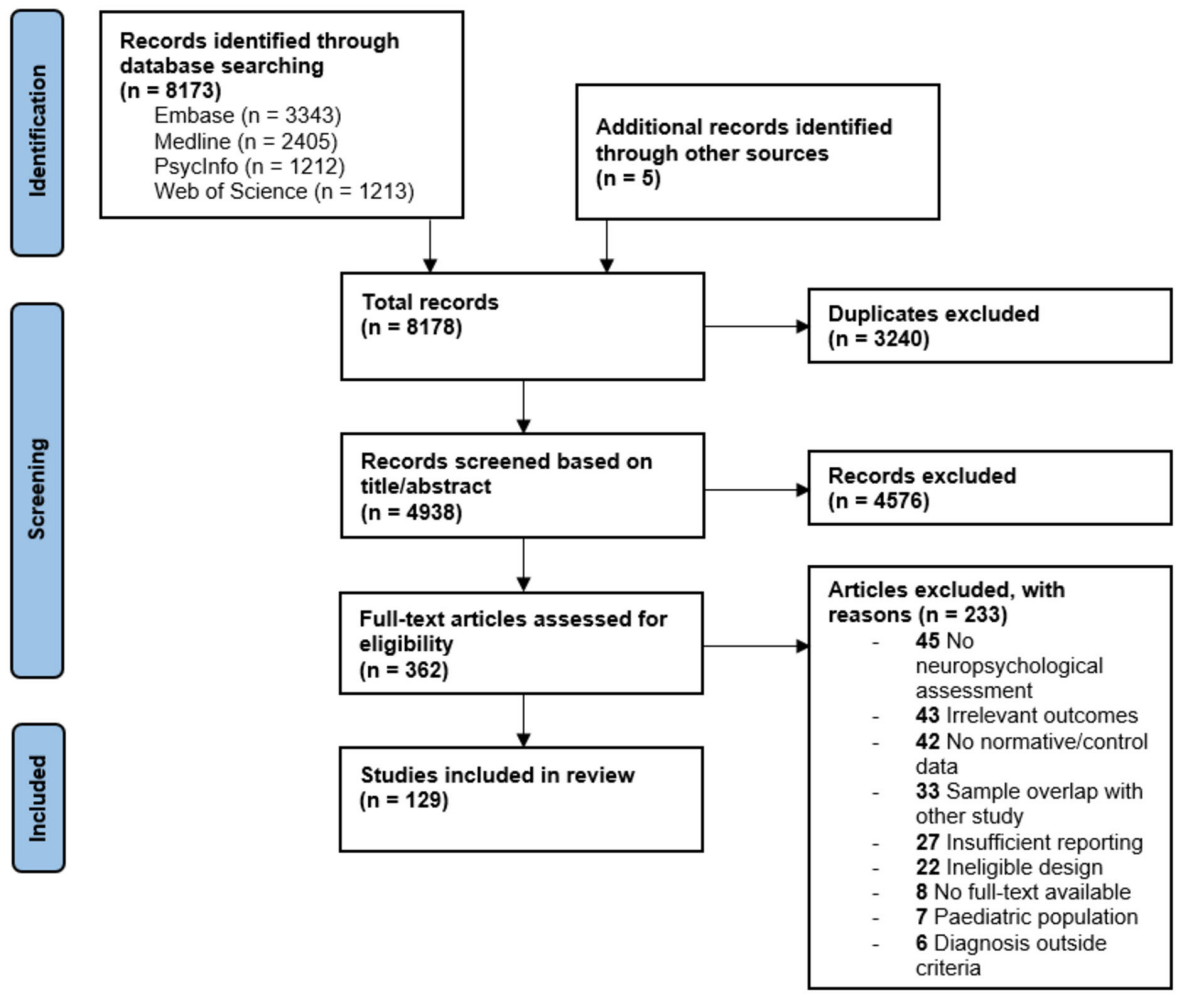
Flowchart of included articles for this review

Our search in Embase, MEDLINE, PsycInfo, and Web of Science provided a total of 8,173 articles, and five additional articles were identified through manual search. Of the initial 8,178 articles, 362 were eventually full-text screened and 129 were included in this review ultimately [1, 29–156]. Details about the inclusion and exclusion of articles in this review are shown in the PRISMA flowchart in Fig. 1.

### Study characteristics

A total of 3,140 patients with cerebellar diseases from the 129 included articles were included in this review. The majority of patients (*n* = 2,252 from *k* = 91) had a degenerative cerebellar disorder. At least 990 patients had spinocerebellar ataxia, 387 patients had a recessive ataxia, and 193 patients had MSA-C (not all included papers specified the exact ataxia type, or types were taken together). A smaller proportion of all patients (*n* = 773 from *k* = 36) had isolated focal lesions (surgical, ischemic, or hemorrhagic) in the cerebellum. The remaining 115 patients came from four studies in which these etiologies were mixed [1, 42, 59, 82]. Two studies included mixed etiologies, but reported outcomes separately for the focal and degenerative groups [101, 108]. The mean age of the patients ranged between 27.0 and 68.2 years for all studies, with an average of 49.9 years. The proportion of men ranged from 20 to 100%. Details regarding characteristics per study are provided in Supplementary Information 4. Most studies (*k* = 111) used control data of healthy participants (*n* = 2,644), and the other studies reported standardized outcomes using reference values of a control cohort (*k* = 18). In 75 studies (58%), the control/standardized data were matched/corrected for both age, sex, and education. Included studied were conducted in 27 different countries, but most were from Germany (*k* = 21) and USA (*k* = 20). Studies were published between 1984 and 2024. A substantial proportion of studies also reported the outcomes of cognitive screeners (MMSE: *k* = 48 and MoCA: *k* = 9). Of the studies reporting on MMSE scores, only two studies reported scores below the cut-off (24 points) indicative of (severe) cognitive dysfunction. Of the studies reporting MoCA scores, only one study reported scores below the cut-off (26 points) indicative of mild cognitive impairment.

### Quality assessment

The results of the quality assessment of all included studies can be found in Supplementary Information 3. Quality of participant selection was overall good, of the 129 studies only two did not report inclusion criteria. Five studies did not report disease characteristics, and most studies did not or not completely report the appropriateness of data assessment (e.g., whether standardized testing was performed by trained assessors). Quality of the outcome measures was overall good (except for one study) with cognitive tests and normative data well specified. Cognitive assessment was appropriate, but not all studies corrected or matched outcomes for both age, sex, and education level. Only a few studies reported performance bias (*k* = 4), by indicating no or low risk of underperformance. Attrition bias was reported in nearly half of the studies (*k* = 62). Possible confounders, such as motor impairment and dysarthria, were commonly described and presented no risk in 68 studies (52%) and a low risk in 33 studies (26%). In the remaining 28 studies (22%), this was not described.

### Cognitive impairment

**Fig. 2:**
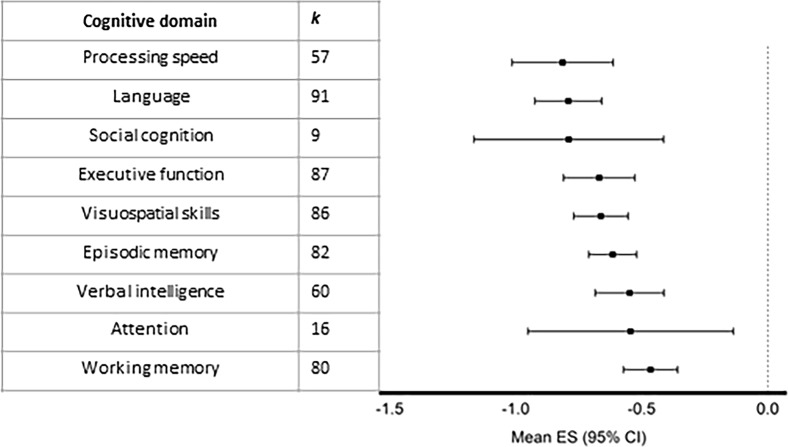
Effect sizes with 95% CI per cognitive domain for all cerebellar etiologies

Figure 2 shows the results of the meta-analysis, patients with cerebellar disorders from all etiologies demonstrated significantly worse performance compared to controls/normative data in all cognitive domains (for more details regarding separate cognitive domains, see Supplementary Information 5). The initially described features of CCAS (i.e., language, executive function, and visuospatial skills) were the most commonly tested domains. However, the largest effect sizes were reported for processing speed (ES [95% CI] = − 0.83 [−1.04, − 0.63]), language (ES [95% CI] = − 0.81 [−0.94, −0.67]), and social cognition (ES [95% CI] = − 0.81 [− 1.19, − 0.42]). Moderate effect sizes were found for executive function (ES [95% CI] = − 0.68 [− 0.83,− 0.54]), visuospatial skills (ES [95% CI] = − 0.68 [− 0.79,− 0.57]), episodic memory (ES [95% CI] = − 0.64 [− 0.74,− 0.54]), verbal intelligence (ES [95% CI] = −0.56 [− 0.70,− 0.42]), and attention (ES [95% CI] = − 0.56 [− 0.97,− 0.14]). Small effect sizes were reported for the working memory domain (ES [95% CI] = − 0.48 [− 0.58, − 0.37]). All significance levels were below 0.0086.

Heterogeneity measures and indicators for publication bias are provided for each cognitive domain in Table 1. Heterogeneity in terms of I^2^ was variable, but high for the majority of cognitive domains. The social cognition and attention domain had small fail-safe *N* numbers, indicating that the meta-analytic results are likely less robust to publication bias. The Egger’s regression test for funnel plot asymmetry (see Supplementary Information 5) indicated potential publication bias for language and executive function.

**Table 1:** Heterogeneity measures and publication bias indicates per cognitive domain

Cognitive domain	I^2^	τ^2^	Fail-safe *N*	Egger’s test, *p* value
Processing speed	89.2%	0.51	750	0.4747
Language	82.5%	0.33	2779	< 0.0001
Social cognition	81.8%	0.27	20	0.3203
Executive function	80.8%	0.35	1603	0.0370
Visuospatial skills	71.8%	0.17	2532	0.2278
Episodic memory	56.9%	0.11	2880	0.0835
Verbal intelligence	67.9%	0.19	774	0.2524
Attention	86.0%	0.58	11	0.7670
Working memory	65.3%	0.15	1236	0.6263

Effect sizes per subdomain were explored as well. For language, word production had the largest effect size [95% CI] = − 0.91 [− 1.05, − 0.76] (*p* < 0.0001, *k* = 81). The effect sizes for naming, comprehension, and reading were small (− 0.43 [− 0.62, − 0.25], − 0.27 [− 0.49, − 0.06], and − 0.24 [− 1.38, 0.91], respectively). For social cognition, theory of mind had a large effect size [95% CI] = − 0.83 [− 0.42, − 0.24] (*p* = 0.0056, *k* = 6), whereas emotion recognition had a small effect size (− 0.49 [− 0.90, − 0.08]). Processing speed, verbal intelligence, and working memory had no subdomains. Effect sizes of all subdomains are listed in Supplementary Information 6.

### Sub-analysis focal vs. degenerative group

**Fig. 3:**
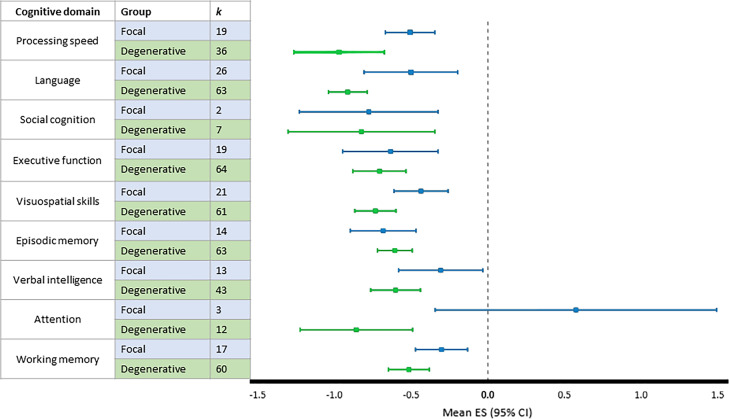
Effect sizes with 95% CI per cognitive domain for both groups (focal vs. degenerative) separately

Outcomes were analyzed for patients with focal cerebellar lesions and degenerative cerebellar disorders separately, as visualized in Fig. 3. Visual inspection reveals distinct magnitudes of cognitive deficits between the groups for several domains (i.e., processing speed, language, visuospatial skills, and verbal intelligence), and cognitive performance was mostly worse in the degenerative group compared to the focal group. Effect sizes for both groups were analyzed in comparison to control/standardized data. Considering patients with focal cerebellar lesions, the largest effect size was observed for social cognition (ES [95% CI] = − 0.78 [− 1.23, − 0.32]). The focal group had a moderate, positive effect size for attention (ES [95% CI] = 0.58 [− 0.34, 1.49]), indicating better performance than control/standardized data. However, for both social cognition and attention, the number of studies was low (*k* = 2 and 3, respectively). All significance levels were below 0.0013, except for verbal intelligence (*p* = 0.0284) and attention (*p* = 0.2197) in the focal group.

## Discussion

This meta-analysis examined the available evidence in the literature on cognitive impairment/functioning in cerebellar disorders. Overall, a substantial amount of studies has been performed in various cerebellar etiologies, of which 129 studies with a total of 3,140 cerebellar patients were eligible for inclusion.

Patients with cerebellar disorders performed significantly worse compared to control or reference groups on all cognitive domains. The deficits were most pronounced in the domains of processing speed, language (particularly word generation), and social cognition (especially theory of mind). Notably, deficits in information processing speed and social cognition were not included in the initial characterization of CCAS by Schmahmann and Sherman (1998), but our meta-analysis shows these to be among the most affected cognitive domains. These domains are neither explicitly incorporated into the CCAS scale, which is a widely used cognitive screener in patients with cerebellar disorders [82]. Cerebellar regions are suggested to play a role in cognitive processing speed through cerebello-cortical connectivity loops, as indicated by functional neuroimaging studies in healthy subjects [157, 158]. The observed language impairment is in agreement with the consensus reached among experts that the cerebellum is involved in linguistic processes, based on clinical, experimental, and neuro-anatomical evidence [159]. In this meta-analysis, we show that especially word production had the largest effect size, which is most commonly assessed with verbal fluency tests (see Supplemental Information 2). It must be noted that selective publication bias of studies showing impairments in the language domain is possible as indicated by the statistically significant Egger’s test. Publication bias might as well be present for our results on social cognition, due to the small number of studies to date. Although we found a large and statistically significant effect size in this domain, this result has large confidence intervals. More research should be performed to gain more insight into the extent of socio-cognitive impairment in cerebellar disorders. Social cognition was initially not associated with cerebellar disorders, but deficits in this domain—both in cerebellar disorders and in other brain diseases—have received increased attention in recent years [160]. There is also growing evidence that the cerebellum appears to have a key role in social interactions [17, 161]. Moreover, the cerebellum is suggested to be associated with theory of mind deficits in several neuropsychiatric disorders, such as schizophrenia and autism [52, 162]. More studies into social cognition are, however, required to draw firm conclusions, as our results are based on limited evidence in this domain.

It must be emphasized that the allocation of neuropsychological tests to cognitive domains may differ across studies and are sometimes debated. One important reason for this is that neuropsychological tests are not always process pure in nature and commonly rely on multiple cognitive domains [163]. For example, verbal fluency tests touch both domains of language and executive function. However, we opted for allocation to the language domain, because language processing is regarded as the core component of these tests [164].

Outcomes revealed substantial heterogeneity of study outcomes, indicated by I^2^ values ranging from 65 to 89% across domains. The large variation in outcomes between studies may in part be explained by the different neuropsychological tests used. The challenge of operationalizing cognitive domains also plays a role here, since numerous diverse measures were classified into single domains. To illustrate, all extracted test outcomes from the included studies encompassed 126 different neuropsychological tests, which were ultimately grouped into nine cognitive domains. In addition to the differences in outcome measures, the wide variety of included cerebellar etiologies is also likely to have contributed to the large heterogeneity. Given the large number of potentially relevant variables and the relatively small number of studies, meta-regression to identify those variables that moderated the variability in cognitive outcomes was unfortunately not possible.

Cognitive profiles may differ among the wide range of cerebellar disorders, and therefore, cognitive performance was explored for patients with focal cerebellar lesions and degenerative cerebellar disorders separately. This revealed differences between the groups, varying across the domains. Overall, the degenerative group had larger effect sizes compared to the focal group, indicating worse cognitive performance. The worse performance in the degenerative group might be explained by extracerebellar involvement in these disorders, since many degenerative disorders are not purely cerebellar. For example, in several spinocerebellar ataxia subtypes (e.g., SCA1, 2, 3, and 7), more extensive cognitive deficits can also arise from corticostriatal disruption, beyond the cerebello-cortical loops [165]. Furthermore, several studies in this meta-analysis have directly compared various types of degenerative cerebellar disorders and reported notable differences [41, 47, 67, 93, 100, 106, 115]. Since cognitive profiles may even differ between SCA subtypes, and our primary aim was to provide a comprehensive overview of cognitive impairment in any type of cerebellar disease, we did not opt for more subdivisions in our analyses. Patients with degenerative disorders may also have reduced cognitive function due to living with a progressive neurological condition, as both the physical burden of their condition (i.e., disabilities in activities of daily living) and the mental burden of having a progressive disease can exacerbate cognitive symptoms [166].

Bias in the results of the included studies may have arisen due to the presence of motor symptoms (e.g., upper limb, articulatory, and visual difficulties). Possible confounding due to these symptoms was commonly reported in the included studies, but could nevertheless have influenced the outcomes. Dysarthria may, for instance, have influenced verbal fluency test outcomes in the language domain. To account for dysarthria, a correction based on the person’s articulation speed has been proposed [167], but this has not been applied in most studies. A similar approach could be employed for upper limb difficulties through correction by a measure of manual dexterity [167]. To tackle the issue of motor slowness in timed neuropsychological tests, recommendations in the selection of tests with minimal motor interference have been provided previously [168].

Other potential confounding variables in assessing cognitive function might be neurotropic drug use (e.g., anticholinergics and benzodiazepines) and the presence of comorbidities, these were mostly reported as exclusion criteria in studies [169]. However, for patients with focal lesions, half of the included studies did not provide sufficient detail about the reliability of the isolated lesions (see Supplementary Information 3). This raises uncertainty about whether supratentorial lesions were excluded and if the lesions were indeed confined to the cerebellum. Affective symptoms (e.g., anxiety and depression) are not often reported as exclusion criteria, but are common in cerebellar disorders and part of the affective component of CCAS [170]. Although this was outside the scope of our meta-analysis, affect is a critical aspect of CCAS, as the cerebellum also plays a role in emotional processes [3, 171]. The close interplay between affect and cognition makes that affective symptoms can influence cognitive outcomes and vice versa [172]. Another factor influencing neuropsychological results is age, as higher age may contribute to worse cognitive performance. However, 123 of the 129 studies (95%) matched or corrected their control or normative data for age. Additionally, most studies (108 out of 129, 84%) also matched or corrected their outcomes for education level and/or IQ. Finally, cognitive results may be affected by possible radiation therapy in patients with a cerebellar tumor [173]. Of the five studies involving tumors, one reported the use of radiation therapy and the remaining did not provide information on this. Excluding these five studies from our meta-analysis had little-to-no impact on the effect sizes across cognitive domains, except for processing speed (−0.87 [−1.09, −0.65], *p* < 0.0001, *k* = 53) and attention (−0.67 [−1.09, −0.25], *p* = 0.0017, *k* = 14), which showed larger effect sizes.

Some limitations need to be addressed. First, we were unable to analyses outcomes on the individual level of patients, since the majority of papers we included in this meta-analysis only reported data of neuropsychological outcomes on a group level. Second, the course of cognitive performance over time has not been taken into account. Also, the time to neuropsychological assessment after focal lesions varied, as well as the disease duration in the degenerative group. A heterogeneous course of cognitive function is presumed after cerebellar strokes, and for degenerative disorders, a decline could be expected due to the progressive disease course [116]. Future studies with longitudinal designs may provide more clarity about the impact of this on cognitive outcomes. Finally, even focal lesions may not be truly isolated in the cerebellum. We have taken this into account when selecting studies and we have included this in the quality assessment, but this aspect was not extensively described in all studies. Therefore, we cannot rule out the presence of supratentorial lesions.

To conclude, cerebellar disorders can impact many cognitive domains, extending beyond the initial description of Schmahmann and Sherman (1998), which was primarily focused on executive functioning, visuospatial skills, and language. Health care professionals should be aware of the wide range of cognitive problems that can occur in patients with cerebellar disorders, which may interfere with daily living. Our results consider a heterogeneous group of cerebellar etiologies and indicate that impairments were most common for processing speed, language (word generation), and social cognition (theory of mind). In all, this is the first study to meta-analyze the cognitive outcomes in cerebellar disorders and as such contributes to the understanding of the cerebellum's role in cognition. Its results also shed light on the cognitive deficits experienced by individuals with cerebellar disorders, which may have a large impact on daily life, and they could help to pave the way for more targeted interventions and therapeutic strategies.

## Supplementary Information

Below is the link to the electronic supplementary material.

Supplementary file1 (DOCX 28 KB)

Supplementary file2 (DOCX 37 KB)

Supplementary file3 (DOCX 105 KB)

Supplementary file4 (DOCX 87 KB)

Supplementary file5 (DOCX 3068 KB)

Supplementary file6 (DOCX 16 KB)
