# Supplementary material for: Cognition in cerebellar disorders: What’s in the profile? A systematic review and meta-analysis
Source: J Neurol. 2025 Mar 6;272(3):250. doi: 10.1007/s00415-025-12967-8 (PMC11885410; doi:10.1007/s00415-025-12967-8)
Supplement: Supplementary file 1 — Supplementary file1 (DOCX 28 KB) [file 415_2025_12967_MOESM1_ESM.docx]

# **Search strategy**

Databases: Embase, MEDLINE, PsycINFO en Web of Science.

| Ovid MEDLINE(R) ALL <1946 to July 16, 2024> | | |
| --- | --- | --- |
|  |  | Hits (no.) |
| 1 | (exp Cerebellar Diseases/ or (Cerebel*.ti,ab,kf. Adj3 (exp Stroke/ or hemorrhage*.ti,ab,kf. or haemorrhage*.ti,ab,kf. or tumor*.ti,ab,kf. or tumour*.ti,ab,kf. or stroke*.ti,ab,kf. or infarct*.ti,ab,kf. or bleed*.ti,ab,kf. or Ataxia*.ti,ab,kf. or degenerati*.ti,ab,kf. or atroph*.ti,ab,kf. or genetic* .ti,ab,kf. Or injury .ti,ab,kf.)) or Machado-Joseph disease*.ti,ab,kf. or spinocerebellar ataxia*.ti,ab,kf. or Friedreich.ti,ab,kf.) and (exp Cognition/ or Cognitive Dysfunction/ or exp Memory/ or exp Attention/ or exp Executive Function/ or cognit*.ti,ab,kf. or language.ti,ab,kf. or CCAS.ti,ab,kf. or memory.ti,ab,kf. or attention.ti,ab,kf. Or visuospatial.ti,ab,kf. or visuoconstruction.ti,ab,kf. or executive.ti,ab,kf. or emotion*.ti,ab,kf. or social.ti,ab,kf. or neurocognit*.ti,ab,kf. or neuropsycholog*.ti,ab,kf. or processing speed.ti,ab,kf.) | 4090 |
| 2 | exp animals/ not humans.sh. | 5240699 |
| 3 | 1 not 2 | 3914 |
| 4 | (mice OR mouse OR murine OR rat* OR animal* OR Child* OR infant* OR pediatr* OR teen*).ti. | 3350841 |
| 5 | 3 not 4 | 3397 |
| 6 | (review or case report or meta-analysis).ti. | 1119887 |
| 7 | 5 not 6 | 3061 |
| 8 | "Review" [Publication Type] | 4243305 |
| 9 | 7 not 8 | 2405 |

| Embase <1974 to 2024 July 16> | | |
| --- | --- | --- |
|  |  |  |
| 1 | (exp cerebellar ataxia/ or exp cerebellum degeneration/ or cerebellum hemorrhage/ or cerebellum infarction/ or cerebellum injury/ or exp cerebellum tumor/ or spinocerebellar degeneration/ or (Cerebel*.ti,ab,kf. Adj3 (exp cerebrovascular accident/ or hemorrhage*.ti,ab,kf. or haemorrhage*.ti,ab,kf. or tumor*.ti,ab,kf. or tumour*.ti,ab,kf. or stroke*.ti,ab,kf. or infarct*.ti,ab,kf. or bleed*.ti,ab,kf. or Ataxia*.ti,ab,kf. or degenerat*.ti,ab,kf. or atroph*.ti,ab,kf. or genetic* .ti,ab,kf. Or injury .ti,ab,kf.)) or Machado-Joseph disease*.ti,ab,kf. or spinocerebellar ataxia*.ti,ab,kf. or Friedreich.ti,ab,kf.) and (executive function/ or memory/ or alertness/ or cognit*.ti,ab,kf. or language.ti,ab,kf. or CCAS.ti,ab,kf. or memory.ti,ab,kf. or attention.ti,ab,kf. or visuospatial.ti,ab,kf. or visuoconstruction.ti,ab,kf. or executive.ti,ab,kf. or emotion*.ti,ab,kf. or social.ti,ab,kf. or neurocognit*.ti,ab,kf. or neuropsycholog*.ti,ab,kf. or processing speed.ti,ab,kf.) | 7839 |
| 2 | ((exp animal/ or nonhuman/) NOT exp human/) | 7372648 |
| 3 | 1 not 2 | 7457 |
| 4 | (mice OR mouse OR murine OR rat* OR animal* OR Child* OR infant* OR pediatr* OR teen*).ti. | 3846333 |
| 5 | 3 not 4 | 6771 |
| 6 | Limit 1 to conference abstract status | 2439 |
| 7 | 5 not 6 | 4616 |
| 8 | (review or case report or meta-analysis).ti. | 1308456 |
| 9 | 7 not 8 | 4114 |
| 10 | exp "conference review"/ or exp "systematic review"/ or exp "review"/ or exp "systematic review (topic)"/ | 3324068 |
| 11 | 9 not 10 | 3343 |

| APA PsycInfo <1806 to July Week 2 2024> | | |
| --- | --- | --- |
|  |  |  |
| 1 | (Ataxia/ or (Cerebel*.ti,ab,id. adj3 (cerebrovascular accidents/ or hemorrhage*.ti,ab,id. or haemorrhage*.ti,ab,id. or tumor*.ti,ab,id. or tumour*.ti,ab,id. or stroke*.ti,ab,id. or infarct*.ti,ab,id. or bleed*.ti,ab,id. or Ataxia*.ti,ab,id. or degenerat*.ti,ab,id. or atroph*.ti,ab,id. or genetic* .ti,ab,id. Or injury .ti,ab,id.)) or Machado-Joseph disease*.ti,ab,id. or spinocerebellar ataxia*.ti,ab,id. or Friedreich.ti,ab,id.) and (Cognition/ or Cognitive Impairment/ or cognit*.ti,ab,id. or language.ti,ab,id. or CCAS.ti,ab,id. or memory.ti,ab,id. or attention.ti,ab,id. or visuospatial.ti,ab,id. or visuoconstruction.mp. or executive.ti,ab,id. or emotion*.ti,ab,id. or social.ti,ab,id. or neurocognit*.ti,ab,id. or neuropsycholog*.ti,ab,id. or processing speed.ti,ab,id.) | 1513 |
| 2 | (mice OR mouse OR murine OR rat* OR animal* OR Child* OR infant* OR pediatr* OR teen*).ti. | 640922 |
| 3 | 1 not 2 | 1360 |
| 4 | (review or case report or meta-analysis).ti. | 212281 |
| 5 | 3 not 4 | 1277 |
| 6 | 5 and "Journal" [Publication Type] | 1212 |

Web of Science = 1213

TS=(("Cerebellar Dis*"

OR

(Cerebel* NEAR/3 (Stroke* OR Hemorrhage* OR haemorrhage* OR tumor* OR tumour* OR infarct* OR bleed* OR Ataxia* OR degenerate* OR atroph* OR genetic* OR injury))

OR ‘’Machado-Joseph disease*’’ OR ‘’spinocerebellar ataxia*’’ OR Friedreich)

AND

(cognit* OR language OR CCAS OR memory OR attention OR visuospatial OR visuoconstruction OR executive OR emotion* OR social OR neurocognit* OR neuropsycholog* OR “processing speed”))

NOT TS=(review OR meta-analysis OR case report OR animal* OR rat* OR mouse OR mice OR murine OR child* OR infant* OR pediatr* OR teen*)

**Additional records identified through other sources: 5**

- Schmahmann JD, Sherman JC. The cerebellar cognitive affective syndrome. Brain. 1998 Apr;121 ( Pt 4):561-79. doi: 10.1093/brain/121.4.561. PMID: 9577385.
- Alexander MP, Gillingham S, Schweizer T, Stuss DT. Cognitive impairments due to focal cerebellar injuries in adults. Cortex. Sep 2012;48(8):980-90. doi:10.1016/j.cortex.2011.03.012
- Corben LA, Delatycki MB, Bradshaw JL, Horne MK, Fahey MC, Churchyard AJ, Georgiou-Karistianis N. Impairment in motor reprogramming in Friedreich ataxia reflecting possible cerebellar dysfunction. J Neurol. 2010 May;257(5):782-91. doi: 10.1007/s00415-009-5410-1. Epub 2009 Dec 3. PMID: 19957189.
- G. Ben-Yehudah and J.A. Fiez, Impact of cerebellar lesions on reading and phonological processing, Ann N Y Acad Sci 1145 (2008), 260–274.
- Clausi S, Olivito G, Siciliano L, Lupo M, Laghi F, Baiocco R, Leggio M. The cerebellum is linked to theory of mind alterations in autism. A direct clinical and MRI comparison between individuals with autism and cerebellar neurodegenerative pathologies. Autism Res. 2021 Nov;14(11):2300-2313. doi: 10.1002/aur.2593. Epub 2021 Aug 10. PMID: 34374492; PMCID: PMC9291804.
