# Supplementary material for: Cognition in cerebellar disorders: What’s in the profile? A systematic review and meta-analysis
Source: J Neurol. 2025 Mar 6;272(3):250. doi: 10.1007/s00415-025-12967-8 (PMC11885410; doi:10.1007/s00415-025-12967-8)
Supplement: Supplementary file 2 — Supplementary file2 (DOCX 37 KB) [file 415_2025_12967_MOESM2_ESM.docx]

# **Validated neuropsychological tests**

| **Cognitive domain**  Subdomain | **Test name** | **Test abbreviation** | ***k*** |
| --- | --- | --- | --- |
| **Verbal intelligence** | | | |
|  | Verbal Comprehension Index (Wechsler Adult Intelligence Scale) (incl. Vocabulary, Information/Orientation, Similarities, Comprehension) | WAIS-VCI | 32 |
|  | IQ estimates (Wechsler Adult Intelligence Scale) (incl. verbal, performance, global, full-scale, spatial) | WAIS-IQ | 21 |
|  | Form Board Test | FBT | 1 |
|  | National Adult Reading Test | NART | 4 |
|  | North American Adult Reading Test | NAART | 3 |
|  | International Reading Speed test | IRS | 1 |
|  | Peabody Picture Vocabulary test | PPV | 3 |
|  | Multiple Choice Vocabulary test (Mehrfachwahl Wortschatztest) | MCV | 6 |
|  | Wide Range Achievement Test | WRAT | 1 |
|  | Mill-Hill B Vocabulary test | MHBV | 1 |
| **Visuospatial skills** | | | |
| Visuospatial reasoning/ construction | Perceptual Reasoning Index (Wechsler Adult Intelligence Scale) (incl. Block Design, Picture Completion, Matrix Reasoning, Visual Puzzles, Object Assembly) | WAIS-PRI | 31 |
|  | Hooper Visual Organization Test | HVOT | 9 |
|  | Raven’s Progressive Matrices | RPM | 21 |
|  | Picture Arrangement (Wechsler Adult Intelligence Scale) | WAIS-PA | 10 |
|  | Rey-Osterrieth Complex Figure - copy | ROCF-copy | 25 |
|  | Complex Figure Test - copy | CFT-copy | 3 |
|  | Rybakoff's Figures Test | RFT | 1 |
|  | Five-Point Test | FPT | 3 |
|  | Clock Drawing Test | CDT | 8 |
|  | Mental Folding Test | MFT | 3 |
|  | Cube Rotation Test | CRT | 1 |
|  | Drawing Test (incl. Cube, Pentagon, Star) | DT | 4 |
|  | Bender-Gestalt Test | BGT | 1 |
|  | Constructional Praxis | CP | 1 |
|  | Figural Fluency / Design fluency (DKEFS) | FF | 3 |
|  | Counting of Surfaces (Leistungsprüfserie) | LPS-CS | 1 |
|  | Identification of Rules (Leistungsprüfserie) | LPS-IR | 1 |
| Perception | Judgment of Line Orientation | JLO | 13 |
|  | Line Bisection | LB | 3 |
|  | Visual Object and Space Perception | VOSP | 1 |
|  | Benton Facial Recognition test | BFR | 7 |
| Visual scanning | Letter Cancellation Test | LCT | 2 |
|  | Visual Scanning (Test of Attentional Performance) | TAP-VS | 3 |
|  | Match to Sample visual search (Cambridge Neuropsychological Test Automated Battery) | CANTAB-MTS | 2 |
|  | Attentional Matrices Test | AMT | 2 |
| **Language** | |  | |
| Word production | Verbal Fluency (Controlled Oral Word Association Test, incl. semantic, phonemic, action verbal fluency) | VF | 80 |
|  | Rapid Verbal Retrieve | RVR | 1 |
|  | Alternate Uses Test | AUT | 1 |
| Naming | Boston Naming Test / Noun-naming test | BNT | 30 |
| Comprehension | Token Test | TT | 9 |
| Reading | Woodcock Reading Mastery Test | WRMT | 1 |
|  | Nelson–Denny Comprehension Test | NDCT | 1 |
|  | Aachen Aphasia Test | AAT | 1 |
|  | Gülhane Aphasia Test | GAT | 1 |
|  | Neuropsychological Examination for Aphasia (Esame Neuropsicologico dell’Afasia) | ENPA | 1 |
|  | Neurosensory Centre Comprehensive Examination of Aphasia | NCCEA | 1 |
|  | Test of Language Competence | TLC | 1 |
|  | The Word Test | TWT | 1 |
| **Attention** | |  | |
| Alertness | Alertness (Test of Attentional Performance) | TAP-A | 2 |
| Sustained attention | Digit Vigilance Test | DVT | 1 |
|  | Continuous Performance Test | CPT | 4 |
|  | Auditory’ s Vigilance | AV | 2 |
| Divided attention/ dual-tasking | Paced Auditory Serial Addition Test | PASAT | 5 |
|  | Divided attention (Test of Attentional Performance) | TAP-DA | 1 |
|  | Mental Control (Wechsler Memory Scale) | WMS-MC | 2 |
| **Executive function** | |  | |
| Response inhibition | Stroop Colour-Word Test card III | SCWT-III | 8 |
|  | Stroop Colour-Word Test Interference | SCWT-int | 34 |
|  | Go-NoGo (Test of Attentional Performance) | TAP-GO | 2 |
|  | Rule Shift Cards test (Behavioural Assessment of the Dysexecutive Syndrome) | BADS-RSC | 2 |
|  | Behavioural Dyscontrol Scale | BDS | 2 |
|  | Hayling Sentence Completion test | HSC | 3 |
| Shifting | Trail Making Test - B | TMT-B | 34 |
|  | Trial Making Test – B-A or B/A | TMT-BA | 17 |
|  | Wisconsin Card Sorting Test | WCST | 38 |
|  | Brixton Spatial Anticipation Test | BSAT | 3 |
|  | Switching Categories | SC | 9 |
|  | Intra-Extra Dimensional Set Shift (Cambridge Neuropsychological Test Automated Battery) | CANTAB-IED | 3 |
|  | Big/Little Circle (Cambridge Neuropsychological Test Automated Battery) | CANTAB-BLC | 1 |
| Planning | Tower of London test | TL | 3 |
|  | Tower of Hanoi | TH | 4 |
|  | Zoo Map (Behavioural Assessment of the Dysexecutive Syndrome) | BADS-map | 2 |
|  | Six elements (Behavioural Assessment of the Dysexecutive Syndrome) | BADS-SE | 1 |
|  | Porteus Maze test | PM | 1 |
|  | Stockings of Cambridge (Cambridge Neuropsychological Test Automated Battery) | CANTAB-SOC | 2 |
| **Working memory** | |  | |
|  | Digit Span (incl. Forward and Backward) | DS | 61 |
|  | Spatial Span | SS | 8 |
|  | Visual Span | VS | 4 |
|  | Listening Span | LS | 2 |
|  | Memory for Sentences (Stanford Binet (4th) Intelligence Scale) | SBIS-MS | 1 |
|  | Corsi Block-Tapping test / Corsi Span | CBT | 13 |
|  | Working Memory Index (Wechsler Adult Intelligence Scale) (incl. Letter-Number Sequencing, Arithmetic) | WAIS-WMI | 25 |
|  | Working Memory (Test of Attentional Performance) | TAP-WM | 5 |
|  | Spatial Working Memory (Cambridge Neuropsychological Test Automated Battery) | CANTAB-SWM | 1 |
| **Episodic memory** | |  | |
| Memory scale immediate | Immediate Memory Index (Wechsler Memory Scale) (incl. Logical Memory I, Visual Paired Associates I, Visual Reproduction I, Family Pictures) | WMS-IMI | 25 |
|  | Post Graduate Institute Memory Scale - immediate recall | PGIMS-imm | 1 |
| Immediate verbal learning and memory | Rey Auditory Verbal Learning Test – immediate recall | RAVLT-imm | 23 |
|  | California Verbal Learning Test – immediate recall | CVLT-imm | 8 |
|  | Hopkins Verbal Learning Test – immediate recall | HVLT-imm | 4 |
|  | Seoul Verbal Learning Test – immediate recall | SVLT-imm | 3 |
|  | -Other- Learning test - immediate | OLT-imm | 8 |
|  | Selective Reminding Test – immediate recall | SRT-imm | 3 |
|  | Paired Associate Word Learning test - immediate | PAWL-imm | 5 |
| Immediate visuospatial learning and memory | Rey-Osterrieth Complex Figure – immediate recall | ROCF-imm | 21 |
|  | Complex Figure Test - immediate | CFT-imm | 1 |
|  | 10/36 Spatial Recall Test - immediate | SPART-imm | 5 |
|  | Recurring Figures | RF | 4 |
|  | Brief Visuospatial Memory Test - immediate | BVMT-imm | 1 |
|  | Benton Visual Retention Test | BVRT | 7 |
|  | Paired Associates Learning (Cambridge Neuropsychological Test Automated Battery) | CANTAB-PAL | 1 |
| Memory scale delayed | Delayed Memory Index (Wechsler Memory Scale) (incl. Logical Memory II, Visual Paired Associates II, Visual Reproduction II) | WMS-DMI | 24 |
|  | Post Graduate Institute Memory Scale – delayed recall | PGIMS-del | 1 |
| Delayed verbal learning and memory | Rey Auditory Verbal Learning Test – delayed recall or recognition | RAVLT-del | 19 |
|  | California Verbal Learning Test – delayed recall | CVLT-del | 9 |
|  | Hopkins Verbal Learning Test – delayed recall | HVLT-del | 3 |
|  | Seoul Verbal Learning Test – delayed recall | SVLT-del | 3 |
|  | -Other- Learning test - delayed | OLT-del | 9 |
|  | Paired Associate Word Learning test - delayed | PAWL-del | 2 |
|  | Selective Reminding Test – delayed recall | SRT-del | 3 |
| Delayed visuospatial learning and memory | Rey-Osterrieth Complex Figure – delayed recall | ROCF-del | 16 |
|  | Complex Figure Test - delayed | CFT-del | 2 |
|  | 10/36 Spatial Recall Test - delayed | SPART-del | 5 |
|  | Brief Visuospatial Memory Test - delayed | BVMT-del | 1 |
|  | Story Recall test | SR | 6 |
|  | Visual memory or verbal memory or auditory memory or memory quotient (Wechsler Memory Scale) | WMS | 6 |
| **Processing speed** | |  | |
|  | Stroop Colour-Word Test card I | SCWT-I | 23 |
|  | Stroop Colour-Word Test card II | SCWT-II | 22 |
|  | Symbol Digit Modalities Test | SDMT | 8 |
|  | Processing Speed Index (Wechsler Adult Intelligence Scale) (incl. Digit Symbol-Coding, Symbol Search) | WAIS-PSI | 14 |
|  | Trail Making Test - A | TMT-A | 37 |
| **Social cognition** | |  | |
| Emotion recognition | Facial Expressions of Emotion – Stimuli and Tests | FEEST | 1 |
|  | Vocal Emotional Prosody Recognition task | VEPR | 1 |
|  | Geneva Emotion Recognition Test | GERT | 1 |
|  | Ekman Facial Expressions | EFE | 1 |
| Theory of mind | Reading the Mind in the Eyes Test | RMET | 3 |
|  | Happé's Strange Stories | HSS | 1 |
|  | Faux Pas test | FP | 4 |
