# Supplementary material for: Cognition in cerebellar disorders: What’s in the profile? A systematic review and meta-analysis
Source: J Neurol. 2025 Mar 6;272(3):250. doi: 10.1007/s00415-025-12967-8 (PMC11885410; doi:10.1007/s00415-025-12967-8)
Supplement: Supplementary file 3 — Supplementary file3 (DOCX 105 KB) [file 415_2025_12967_MOESM3_ESM.docx]

# **Quality assessment**

All included articles were assessed in terms of quality and risk of bias, and scored as follows:

**Ο** No/low concerns

**Ο** Some concerns

**Ο** High concerns
X Unknown

Quality of participant selection

1. Criteria for inclusion

**Ο** Present and clearly defined

**Ο** Partly present, narrative description

**Ο** Absent

1. Demographic characteristics of participants (sex, age, education)

**Ο** Present and adequately described, all present

**Ο** Partly present: at least one item mentioned

**Ο** Absent

1. Specification of diagnosis (subtype, genetic/imaging confirmation, description lesion)

**Ο** Present and clear diagnosis

**Ο** Partly present, information is sufficient but not extensive

**Ο** Absent, no or very little information specified

1. [Focal lesions group]
   Reliability of isolated lesions to the cerebellum (are supratentorial lesions described/excluded, presumed other influences on non-cerebellar structures (e.g. radiation therapy in tumour)

**Ο** Clearly described and isolated to the cerebellum

**Ο** Narrative description or not completely isolated to the cerebellum

**Ο** Described, but not isolated to the cerebellum

X Unclear, no description

Quality of data collection

1. Disease characteristics (cerebellar disease type, age at onset, disease duration)

**Ο** Present and adequately described, all present

**Ο** Partly present: at least two items mentioned

**Ο** Absent

1. Appropriateness of data collection / assessment (trained assessors, standardized testing)

**Ο** Appropriate: trained assessors and standardized testing

**Ο** Partly appropriate (one item)

**Ο** Not appropriate

X Unclear, not described

Quality of the outcome

1. Specification of cognitive assessment (test and source of normative data)

**Ο** Present and clearly specified: precise information

**Ο** Partly present: reference given for test use, but no further information

**Ο** Absent: no or very little information specified

1. Appropriateness of assessment; was cognitive performance measured in a valid and reliable way / were cognitive outcomes corrected for age, gender, and education level?

**Ο** Appropriate: validated measure for specific cognitive domains

**Ο** Partly appropriate: validated measure, but not for a specific cognitive domain

**Ο** Not appropriate: non-validated measure or not clearly specified

Risk of bias

1. Performance bias; are there reasons for underperformance, is this taken into account with e.g. performance validity measures?

**Ο** No risk, clearly described or taken into account

**Ο** Low risk

**Ο** High risk, expected to affect the results

X Unclear, not described

1. Attrition bias; are exclusions/missing data described and handled accurately?

**Ο** No risk, clearly described and handled accurately

**Ο** Low risk

**Ο** High risk, expected to affect the results

X Unclear, not described

1. Confounders; are possible confounders described? (e.g., motor impairment, dysarthria)

**Ο** No risk, clearly described

**Ο** Low risk

**Ο** High risk, expected to affect the results

X Unclear, not described

## Quality assessment results

| **ID** | **First author(s)** | **Year** | **Quality of**  **participant selection** | | | | **Quality of**  **data collection** | | **Quality of**  **the outcome** | | **Risk of Bias** | | |
| --- | --- | --- | --- | --- | --- | --- | --- | --- | --- | --- | --- | --- | --- |
|  |  |  | **1. Criteria for inclusion** | **2. Demographic characteristics of participants** | **3. Specification of diagnosis** | **4. [Focal lesions group] Reliability of isolated lesions to the cerebellum** | **5. Disease characteristics** | **6. Appropriateness of data collection / assessment** | **7. Specification of cognitive assessment** | **8. Appropriateness of assessment** | **9. Performance bias** | **10. Attrition bias** | **11. Confounders** |
| 1 | Agarwal & Kaur | 2021 | **Ο** | **Ο** | **Ο** |  | **Ο** | **Ο** | **Ο** | **Ο** | x | x | **Ο** |
| 2 | Alexander | 2012 | **Ο** | **Ο** | **Ο** | **Ο** | **Ο** | **Ο** | **Ο** | **Ο** | x | **Ο** | **Ο** |
| 3 | Appollonio | 1993 | **Ο** | **Ο** | **Ο** |  | **Ο** | x | **Ο** | **Ο** | x | **Ο** | **Ο** |
| 4 | Arai | 2003 | **Ο** | **Ο** | **Ο** |  | **Ο** | x | **Ο** | **Ο** | x | **Ο** | **Ο** |
| 5 | Arroyo-Anllo | 1998 | **Ο** | **Ο** | **Ο** |  | **Ο** | **Ο** | **Ο** | **Ο** | x | x | **Ο** |
| 6 | Balas | 2010 | **Ο** | **Ο** | **Ο** |  | **Ο** | x | **Ο** | **Ο** | x | **Ο** | **Ο** |
| 7 | Beldarrain | 1997 | **Ο** | **Ο** | **Ο** | **Ο** | **Ο** | **Ο** | **Ο** | **Ο** | x | x | **Ο** |
| 8 | Ben-Yehudah | 2008 | **Ο** | **Ο** | **Ο** | x | **Ο** | **Ο** | **Ο** | **Ο** | x | **Ο** | **Ο** |
| 9 | Berent | 1990 | **Ο** | **Ο** | **Ο** |  | **Ο** | **Ο** | **Ο** | **Ο** | x | x | **Ο** |
| 10 | Berent | 2002 | **Ο** | **Ο** | **Ο** |  | **Ο** | **Ο** | **Ο** | **Ο** | x | **Ο** | **Ο** |
| 11 | Bolceková | 2017 | **Ο** | **Ο** | **Ο** | **Ο** | **Ο** | **Ο** | **Ο** | **Ο** | x | **Ο** | **Ο** |
| 12 | Bolzan | 2024 | **Ο** | **Ο** | **Ο** |  | **Ο** | **Ο** | **Ο** | **Ο** | x | **Ο** | **Ο** |
| 13 | Botez-Marquard | 1993 | **Ο** | **Ο** | **Ο** |  | **Ο** | **Ο** | **Ο** | **Ο** | x | x | **Ο** |
| 14 | Bracke-Tolkmitt | 1989 | **Ο** | **Ο** | **Ο** | **Ο** | **Ο** | **Ο** | **Ο** | **Ο** | x | x | **Ο** |
| 15 | Braga-Neto | 2012 | **Ο** | **Ο** | **Ο** |  | **Ο** | **Ο** | **Ο** | **Ο** | x | x | **Ο** |
| 16 | Brandt | 2004 | **Ο** | **Ο** | **Ο** |  | **Ο** | **Ο** | **Ο** | **Ο** | x | **Ο** | **Ο** |
| 17 | Brega | 2008 | **Ο** | **Ο** | **Ο** |  | **Ο** | **Ο** | **Ο** | **Ο** | x | **Ο** | x |
| 18 | Bürk | 2001 | **Ο** | **Ο** | **Ο** |  | **Ο** | **Ο** | **Ο** | **Ο** | x | x | **Ο** |
| 19 | Bürk | 2003 | **Ο** | **Ο** | **Ο** |  | **Ο** | **Ο** | **Ο** | **Ο** | **Ο** | **Ο** | **Ο** |
| 20 | Bürk | 2006 | **Ο** | **Ο** | **Ο** |  | **Ο** | **Ο** | **Ο** | **Ο** | **Ο** | **Ο** | **Ο** |
| 21 | Chang | 2009 | **Ο** | **Ο** | **Ο** |  | **Ο** | x | **Ο** | **Ο** | x | x | **Ο** |
| 22 | Chirino | 2018 | **Ο** | **Ο** | **Ο** |  | **Ο** | x | **Ο** | **Ο** | x | **Ο** | **Ο** |
| 23 | Chirino-Pérez | 2021 | **Ο** | **Ο** | **Ο** |  | **Ο** | x | **Ο** | **Ο** | x | **Ο** | **Ο** |
| 24 | Clausi | 2021 | **Ο** | **Ο** | **Ο** |  | **Ο** | **Ο** | **Ο** | **Ο** | x | x | **Ο** |
| 25 | Cocozza | 2018 | **Ο** | **Ο** | **Ο** |  | **Ο** | x | **Ο** | **Ο** | x | x | **Ο** |
| 26 | Cook | 2004 | **Ο** | **Ο** | **Ο** | **Ο** | **Ο** | **Ο** | **Ο** | **Ο** | x | x | x |
| 27 | Cooper | 2010 | **Ο** | **Ο** | **Ο** |  | **Ο** | x | **Ο** | **Ο** | **Ο** | **Ο** | **Ο** |
| 28 | Corben | 2017 | **Ο** | **Ο** | **Ο** |  | **Ο** | **Ο** | **Ο** | **Ο** | x | x | **Ο** |
| 29 | Costabile | 2018 | **Ο** | **Ο** | **Ο** |  | **Ο** | **Ο** | **Ο** | **Ο** | x | x | **Ο** |
| 30 | D'Agata | 2011 | **Ο** | **Ο** | **Ο** |  | **Ο** | **Ο** | **Ο** | **Ο** | x | x | **Ο** |
| 31 | Daum | 1993 | **Ο** | **Ο** | **Ο** | **Ο** | **Ο** | **Ο** | **Ο** | **Ο** | x | **Ο** | **Ο** |
| 32 | de Nóbrega | 2007 | **Ο** | **Ο** | **Ο** |  | **Ο** | x | **Ο** | **Ο** | x | **Ο** | **Ο** |
| 33 | Dimitrov | 1996 | **Ο** | **Ο** | **Ο** |  | **Ο** | **Ο** | **Ο** | **Ο** | x | x | x |
| 34 | Dirnberger | 2010 | **Ο** | **Ο** | **Ο** | **Ο** | **Ο** | x | **Ο** | **Ο** | x | **Ο** | **Ο** |
| 35 | Dogan | 2016 | **Ο** | **Ο** | **Ο** |  | **Ο** | x | **Ο** | **Ο** | x | x | **Ο** |
| 36 | Erdal | 2021 | **Ο** | **Ο** | **Ο** | **Ο** | **Ο** | **Ο** | **Ο** | **Ο** | x | **Ο** | x |
| 37 | Exner | 2004 | **Ο** | **Ο** | **Ο** | x | **Ο** | x | **Ο** | **Ο** | x | x | x |
| 38 | Fan | 2019 | **Ο** | **Ο** | **Ο** | **Ο** | **Ο** | **Ο** | **Ο** | **Ο** | x | **Ο** | x |
| 39 | Fancellu | 2013 | **Ο** | **Ο** | **Ο** |  | **Ο** | **Ο** | **Ο** | **Ο** | x | **Ο** | **Ο** |
| 40 | Fehrenbach | 1984 | **Ο** | **Ο** | **Ο** |  | **Ο** | x | **Ο** | **Ο** | x | x | **Ο** |
| 41 | Feng | 2014 | **Ο** | **Ο** | **Ο** |  | **Ο** | **Ο** | **Ο** | **Ο** | x | **Ο** | **Ο** |
| 42 | Frank | 2010 | **Ο** | **Ο** | **Ο** | **Ο** | **Ο** | **Ο** | **Ο** | **Ο** | x | **Ο** | **Ο** |
| 43 | Frommann | 2012 | **Ο** | **Ο** | **Ο** |  | **Ο** | x | **Ο** | **Ο** | x | x | **Ο** |
| 44 | Gama | 2019 | **Ο** | **Ο** | **Ο** |  | **Ο** | x | **Ο** | **Ο** | x | x | **Ο** |
| 45 | Gambardella | 1998 | **Ο** | **Ο** | **Ο** |  | **Ο** | x | **Ο** | **Ο** | x | **Ο** | x |
| 46 | García | 2022 | **Ο** | **Ο** | **Ο** |  | **Ο** | **Ο** | **Ο** | **Ο** | x | x | **Ο** |
| 47 | Geva | 2021 | **Ο** | **Ο** | **Ο** | **Ο** | **Ο** | **Ο** | **Ο** | **Ο** | x | x | **Ο** |
| 48 | Gigante | 2020 | **Ο** | **Ο** | **Ο** |  | **Ο** | **Ο** | **Ο** | **Ο** | x | x | **Ο** |
| 49 | Globas | 2003 | **Ο** | **Ο** | **Ο** |  | **Ο** | **Ο** | **Ο** | **Ο** | x | **Ο** | x |
| 50 | Gottwald | 2004 | **Ο** | **Ο** | **Ο** | **Ο** | **Ο** | x | **Ο** | **Ο** | x | **Ο** | **Ο** |
| 51 | Harrison | 2019 | **Ο** | **Ο** | **Ο** | **Ο** | **Ο** | **Ο** | **Ο** | **Ο** | x | x | **Ο** |
| 52 | Hart | 1985 | **Ο** | **Ο** | **Ο** |  | **Ο** | x | **Ο** | **Ο** | x | x | **Ο** |
| 53 | Hirono | 1991 | **Ο** | **Ο** | **Ο** |  | **Ο** | **Ο** | **Ο** | **Ο** | x | x | **Ο** |
| 54 | Hoche | 2018 | **Ο** | **Ο** | **Ο** | x | **Ο** | x | **Ο** | **Ο** | x | x | **Ο** |
| 55 | Hokkanen | 2006 | **Ο** | **Ο** | **Ο** | **Ο** | **Ο** | **Ο** | **Ο** | **Ο** | x | **Ο** | **Ο** |
| 56 | Hong | 2011 | **Ο** | **Ο** | **Ο** |  | **Ο** | **Ο** | **Ο** | **Ο** | x | x | x |
| 57 | Jiang | 2013 | **Ο** | **Ο** | **Ο** | **Ο** | **Ο** | x | **Ο** | **Ο** | x | x | **Ο** |
| 58 | Jodzio | 2020 | **Ο** | **Ο** | **Ο** | **Ο** | **Ο** | x | **Ο** | **Ο** | x | x | x |
| 59 | Kansal | 2017 | **Ο** | **Ο** | **Ο** |  | **Ο** | x | **Ο** | **Ο** | x | **Ο** | **Ο** |
| 60 | Karaci | 2008 | **Ο** | **Ο** | **Ο** | **Ο** | **Ο** | **Ο** | **Ο** | **Ο** | x | **Ο** | x |
| 61 | Kawai | 2004 | **Ο** | **Ο** | **Ο** |  | **Ο** | **Ο** | **Ο** | **Ο** | x | x | **Ο** |
| 62 | Kawai | 2008 | **Ο** | **Ο** | **Ο** |  | **Ο** | **Ο** | **Ο** | **Ο** | x | x | **Ο** |
| 63 | Kish | 1988 | **Ο** | **Ο** | **Ο** |  | **Ο** | **Ο** | **Ο** | **Ο** | x | **Ο** | **Ο** |
| 64 | Kish | 1994 | **Ο** | **Ο** | **Ο** |  | **Ο** | **Ο** | **Ο** | **Ο** | x | **Ο** | **Ο** |
| 65 | Klinke | 2010 | **Ο** | **Ο** | **Ο** |  | **Ο** | **Ο** | **Ο** | **Ο** | x | x | **Ο** |
| 66 | Krygier | 2017 | **Ο** | **Ο** | **Ο** |  | **Ο** | x | **Ο** | **Ο** | x | **Ο** | x |
| 67 | Laforce | 2010 | **Ο** | **Ο** | **Ο** |  | **Ο** | **Ο** | **Ο** | **Ο** | x | x | x |
| 68 | Le Pira | 2002 | **Ο** | **Ο** | **Ο** |  | **Ο** | **Ο** | **Ο** | **Ο** | x | x | x |
| 69 | Lee | 2016 | **Ο** | **Ο** | **Ο** |  | **Ο** | x | **Ο** | **Ο** | x | x | x |
| 70 | Lilja | 2005 | **Ο** | **Ο** | **Ο** |  | **Ο** | **Ο** | **Ο** | **Ο** | x | x | **Ο** |
| 71 | Lopes | 2013 | **Ο** | **Ο** | **Ο** |  | **Ο** | x | **Ο** | **Ο** | x | **Ο** | **Ο** |
| 72 | Ma | 2014 | **Ο** | **Ο** | **Ο** |  | **Ο** | **Ο** | **Ο** | **Ο** | x | x | **Ο** |
| 73 | Maddox | 2005 | **Ο** | **Ο** | **Ο** | **Ο** | **Ο** | **Ο** | **Ο** | **Ο** | x | x | **Ο** |
| 74 | Mak | 2016 | **Ο** | **Ο** | **Ο** | x | **Ο** | **Ο** | **Ο** | **Ο** | x | x | **Ο** |
| 75 | Martínez-Regueiro | 2020 | **Ο** | **Ο** | **Ο** |  | **Ο** | **Ο** | **Ο** | **Ο** | x | **Ο** | **Ο** |
| 76 | Maruff | 1996 | **Ο** | **Ο** | **Ο** |  | **Ο** | **Ο** | **Ο** | **Ο** | x | x | **Ο** |
| 77 | Maschke | 2002 | **Ο** | **Ο** | **Ο** |  | **Ο** | x | **Ο** | **Ο** | x | x | **Ο** |
| 78 | Mastammanavar | 2020 | **Ο** | **Ο** | **Ο** |  | **Ο** | x | **Ο** | **Ο** | x | x | x |
| 79 | Meles | 2018 | **Ο** | **Ο** | **Ο** |  | **Ο** | x | **Ο** | **Ο** | x | x | x |
| 80 | Molinari | 2004 | **Ο** | **Ο** | **Ο** | **Ο** | **Ο** | x | **Ο** | **Ο** | x | **Ο** | **Ο** |
| 81 | Moro | 2016 | **Ο** | **Ο** | **Ο** |  | **Ο** | x | **Ο** | **Ο** | x | x | **Ο** |
| 82 | Nachbauer | 2014 | **Ο** | **Ο** | **Ο** |  | **Ο** | **Ο** | **Ο** | **Ο** | x | x | **Ο** |
| 83 | Neau | 2000 | **Ο** | **Ο** | **Ο** | **Ο** | **Ο** | **Ο** | **Ο** | **Ο** | **Ο** | **Ο** | **Ο** |
| 84 | Nieto | 2012 | **Ο** | **Ο** | **Ο** |  | **Ο** | **Ο** | **Ο** | **Ο** | x | **Ο** | **Ο** |
| 85 | Orsi | 2011 | **Ο** | **Ο** | **Ο** |  | **Ο** | **Ο** | **Ο** | **Ο** | x | x | **Ο** |
| 86 | Peterburs | 2010 | **Ο** | **Ο** | **Ο** | **Ο** | **Ο** | x | **Ο** | **Ο** | x | x | **Ο** |
| 87 | Rentiya | 2018 | **Ο** | **Ο** | **Ο** |  | **Ο** | x | **Ο** | **Ο** | x | x | **Ο** |
| 88 | Reumers | 2024 | **Ο** | **Ο** | **Ο** | **Ο** | **Ο** | **Ο** | **Ο** | **Ο** | x | **Ο** | x |
| 89 | Richter | 2004 | **Ο** | **Ο** | **Ο** |  | **Ο** | **Ο** | **Ο** | **Ο** | x | **Ο** | **Ο** |
| 90 | Richter | 2007 | **Ο** | **Ο** | **Ο** | **Ο** | **Ο** | **Ο** | **Ο** | **Ο** | x | x | **Ο** |
| 91 | Rodríguez-Labrada | 2014 | **Ο** | **Ο** | **Ο** |  | **Ο** | x | **Ο** | **Ο** | x | x | **Ο** |
| 92 | Santangelo | 2020 | **Ο** | **Ο** | **Ο** |  | **Ο** | x | **Ο** | **Ο** | x | x | **Ο** |
| 93 | Satoer | 2024 | **Ο** | **Ο** | **Ο** | **Ο** | **Ο** | **Ο** | **Ο** | **Ο** | x | **Ο** | **Ο** |
| 94 | Sayah | 2018 | **Ο** | **Ο** | **Ο** |  | **Ο** | x | **Ο** | **Ο** | x | x | **Ο** |
| 95 | Schmahmann | 1998 | **Ο** | **Ο** | **Ο** | **Ο** | **Ο** | **Ο** | **Ο** | **Ο** | x | **Ο** | **Ο** |
| 96 | Schweizer | 2007 | **Ο** | **Ο** | **Ο** | **Ο** | **Ο** | x | **Ο** | **Ο** | x | x | **Ο** |
| 97 | Shen | 2022 | **Ο** | **Ο** | **Ο** |  | **Ο** | x | **Ο** | **Ο** | x | **Ο** | x |
| 98 | Shin | 2017 | **Ο** | **Ο** | **Ο** | x | **Ο** | x | **Ο** | **Ο** | x | x | x |
| 99 | Shin | 2024 | **Ο** | **Ο** | **Ο** |  | **Ο** | x | **Ο** | **Ο** | x | **Ο** | **Ο** |
| 100 | Shishegar | 2020 | **Ο** | **Ο** | **Ο** |  | **Ο** | x | **Ο** | **Ο** | x | **Ο** | **Ο** |
| 101 | Siciliano | 2022 | **Ο** | **Ο** | **Ο** |  | **Ο** | x | **Ο** | **Ο** | x | **Ο** | **Ο** |
| 102 | Slapik | 2019 | **Ο** | **Ο** | **Ο** |  | **Ο** | **Ο** | **Ο** | **Ο** | x | **Ο** | **Ο** |
| 103 | Starowicz-Filip | 2021 | **Ο** | **Ο** | **Ο** | x | **Ο** | **Ο** | **Ο** | **Ο** | x | **Ο** | **Ο** |
| 104 | Stoodley | 2009 | **Ο** | **Ο** | **Ο** |  | **Ο** | x | **Ο** | **Ο** | x | x | **Ο** |
| 105 | Stoodley | 2016 | **Ο** | **Ο** | **Ο** | **Ο** | **Ο** | **Ο** | **Ο** | **Ο** | x | x | **Ο** |
| 106 | Storey | 1999 | **Ο** | **Ο** | **Ο** |  | **Ο** | x | **Ο** | **Ο** | x | **Ο** | **Ο** |
| 107 | Suenaga | 2008 | **Ο** | **Ο** | **Ο** |  | **Ο** | **Ο** | **Ο** | **Ο** | x | x | **Ο** |
| 108 | Szpisjak | 2017 | **Ο** | **Ο** | **Ο** |  | **Ο** | x | **Ο** | **Ο** | x | **Ο** | x |
| 109 | Tamaš | 2021 | **Ο** | **Ο** | **Ο** |  | **Ο** | **Ο** | **Ο** | **Ο** | x | x | x |
| 110 | Tamura | 2017 | **Ο** | **Ο** | **Ο** |  | **Ο** | x | **Ο** | **Ο** | x | **Ο** | **Ο** |
| 111 | Tamura | 2018 | **Ο** | **Ο** | **Ο** |  | **Ο** | x | **Ο** | **Ο** | x | x | **Ο** |
| 112 | Tanaka | 2003 | **Ο** | **Ο** | **Ο** |  | **Ο** | x | **Ο** | **Ο** | x | x | x |
| 113 | Thomasson | 2019 | **Ο** | **Ο** | **Ο** | **Ο** | **Ο** | **Ο** | **Ο** | **Ο** | x | **Ο** | **Ο** |
| 114 | Timmann | 2004 | **Ο** | **Ο** | **Ο** |  | **Ο** | x | **Ο** | **Ο** | x | x | **Ο** |
| 115 | Torrens | 2008 | **Ο** | **Ο** | **Ο** |  | **Ο** | **Ο** | **Ο** | **Ο** | x | **Ο** | **Ο** |
| 116 | Turner | 2007 | **Ο** | **Ο** | **Ο** | **Ο** | **Ο** | x | **Ο** | **Ο** | x | **Ο** | x |
| 117 | Vaca-Palomares | 2015 | **Ο** | **Ο** | **Ο** |  | **Ο** | **Ο** | **Ο** | **Ο** | x | x | **Ο** |
| 118 | Valis | 2011 | **Ο** | **Ο** | **Ο** |  | **Ο** | x | **Ο** | **Ο** | x | x | **Ο** |
| 119 | van den Berg | 2020 | **Ο** | **Ο** | **Ο** | **Ο** | **Ο** | x | **Ο** | **Ο** | x | **Ο** | x |
| 120 | van der Giessen | 2023 | **Ο** | **Ο** | **Ο** | **Ο** | **Ο** | x | **Ο** | **Ο** | x | **Ο** | **Ο** |
| 121 | Verbitsky | 2023 | **Ο** | **Ο** | **Ο** | **Ο** | **Ο** | x | **Ο** | **Ο** | x | **Ο** | **Ο** |
| 122 | Wallesch | 1990 | **Ο** | **Ο** | **Ο** | x | **Ο** | **Ο** | **Ο** | **Ο** | x | **Ο** | **Ο** |
| 123 | Wang | 2022 | **Ο** | **Ο** | **Ο** | **Ο** | **Ο** | **Ο** | **Ο** | **Ο** | x | **Ο** | x |
| 124 | White | 2000 | **Ο** | **Ο** | **Ο** |  | **Ο** | x | **Ο** | **Ο** | x | x | **Ο** |
| 125 | Witt | 2002 | **Ο** | **Ο** | **Ο** |  | **Ο** | x | **Ο** | **Ο** | x | **Ο** | **Ο** |
| 126 | Wollmann | 2002 | **Ο** | **Ο** | **Ο** |  | **Ο** | **Ο** | **Ο** | **Ο** | x | **Ο** | **Ο** |
| 127 | Yang | 2013 | **Ο** | **Ο** | **Ο** |  | **Ο** | x | **Ο** | **Ο** | x | x | x |
| 128 | Ye | 2023 | **Ο** | **Ο** | **Ο** |  | **Ο** | **Ο** | **Ο** | **Ο** | x | **Ο** | x |
| 129 | Zawacki | 2002 | **Ο** | **Ο** | **Ο** |  | **Ο** | **Ο** | **Ο** | **Ο** | x | **Ο** | **Ο** |
