# Supplementary material for: Cognition in cerebellar disorders: What’s in the profile? A systematic review and meta-analysis
Source: J Neurol. 2025 Mar 6;272(3):250. doi: 10.1007/s00415-025-12967-8 (PMC11885410; doi:10.1007/s00415-025-12967-8)
Supplement: Supplementary file 4 — Supplementary file4 (DOCX 87 KB) [file 415_2025_12967_MOESM4_ESM.docx]

# **Characteristics of included studies**

| **ID** | **First author(s) & year** | **Country** | **Study design** | **N patients & disease type** | **Group** | **Sex, male** | **Age, y** | **Education, y or level** | **Age at onset, y** | **Disease duration or time since lesion, y** | **SARA total score** | **ICARS score** | **Handed-ness, right** | **N controls** | **Control/**  **normative data matched/**  **corrected for:** |
| --- | --- | --- | --- | --- | --- | --- | --- | --- | --- | --- | --- | --- | --- | --- | --- |
| 1 | Agarwal & Kaur, 2021 | India | CSS | 30 SCA12 | D | 20 (67%) | 51.6,  8.0 | 14.3,  2.7 y | 46.3,  8.1 | 5.3,  3.0 |  | 29.8, 12.5 |  | 30 | Age; Sex; Education |
| 2 | Alexander, 2012 | USA & Canada | CSS | 32 cerebellar stroke or benign tumour resection | F | 17 (53%) | 54.78, 14.48 | 13.81, 3.60 y |  | > 3 m |  |  | 27  (84%) | 36 | Age; Sex; Education; Handedness |
| 3 | Appollonio, 1993 | Italy | CSS | 11 cerebellar atrophy | D | 7 (63.6%) | 49.7, 17.6, range: 20-73 | 14.8,  1.9 y | - | 9.6,  9.2 |  |  | 10 (90.9%) | 11 | Sex; Handedness |
| 4 | Arai, 2003 | Japan | CSS | 12 spinocerebellar degeneration | D | 7 (58.3%) | 61.1,  9.0 | 11.4,  3.3 y |  | 13.2,  8.0 |  |  | 12 (100%) | 12 | Age; Sex; Education |
| 5 | Arroyo-Anllo, 1998 | Canada | CSS | 21 OPCA | D | 11 (52%) | 42.91, 11.26 | 8.91, 2.55 y |  |  |  |  |  | 21 | Age; Sex; Education; Handedness |
| 6 | Balas, 2010 | Israel | CSS | 10 MSA-C | D | 6  (60%) | 59.8, 11.8 | 14.0,  2.9 y |  | 3.2,  1.3 |  |  |  | 10 | Age; Sex; Education |
| 7 | Beldarrain, 1997 | Spain | CSS | 26 cerebellar stroke (hematoma and infarction) | F | 18 (69.2%) | 62, range: 31-80 | 8 y |  |  |  |  | 26 (100%) | 16 | Age; Sex; Education |
| 8 | Ben-Yehudah, 2008 | USA | CSS | 6 focal unilateral lesions in cerebellum | F |  | 63.5 | 12.2 y |  | > 3 |  |  |  | 6 | Age; Education |
| 9 | Berent, 1990 | USA | Cohort study | 39 OPCA | D | 17 (44%) | 53,  13.4 | 12.1,  3.3 y |  | 6, range: 1-20 |  |  |  | 25 | Age; Sex; Education |
| 10 | Berent, 2002 | USA | Cohort study | 109 OPCA | D | 51 (47%) | 52.1, 13.0 | 13.1,  3.3 y |  |  |  |  |  | 30 | Age; Sex; Education |
| 11 | Bolceková, 2017 | Czech Republic | CSS | 25 isolated cerebellar lesions | F | 18 (72.0%) | 53.8, 18.6 | 14.4,  3.5 y |  | > 6 m |  | 12.06, 9.68 | 25 (100%) | 25 | Age; Sex; Education |
| 12 | Bolzan, 2024 | Brazil | CSS | 23 SCA3 | D | 8 (34.8%) | 44.92, 8.95 |  |  | 9.17, 4.11 |  |  |  | 58 | Sex; Education |
| 13 | Botez-Marquard, 1993 | Canada | CSS | 30 FA & OPCA | D | 17 (57%) | 39.85, 14.1 | 12.4,  4.2 y |  | 17.4, 7.0, range:  4-30 |  |  | 30 (100%) | 30 | Age; Sex; Education; Handedness |
| 14 | Bracke-Tolkmitt, 1989 | Germany | CSS | 5 cerebellar lesions or atrophy | Both | 4  (80%) | 45.0, 20.86 |  |  | 7.25, 6.86 |  |  |  | 10 | Age; Education |
| 15 | Braga-Neto, 2012 | Brazil | CSS | 38 SCA3 | D | 47% | 42.3, 10.1 | 12.7,  3.0 y | 34.7, 10.1 | 7.3,  4.4 | 13.2, 9.1 | 39.4, 23.7 |  | 31 | Age; Sex; Education |
| 16 | Brandt, 2004 | USA | CSS | 31 primary cerebellar degeneration | D | 14 (45.2%) | 52.0, 12.7 | 14.4,  2.5 level | 40.0, 15.5 |  |  | 40.6, 19.2 | 30 (96.8%) | 29 | Age; Sex; Education |
| 17 | Brega, 2008 | USA | CSS | 47 FXTAS | D | 47 (100%) | 68.2 | 15.4 y |  |  |  |  |  | 41 | Age; Education; Ethnicity |
| 18 | Bürk, 2001 | Germany | CSS | 14 SCA1 | D | 9  (64%) | 48.2, 9.3, range: 35-65 |  | 38.7, 6.1, range: 31-54 | 9.6, 5.1, range:  2-17 |  |  |  | 11 | Age; Sex; IQ |
| 19 | Bürk, 2003 | Germany | CSS | 25 SCA2 & SCA3 | D |  | 47.6, 12.8 |  | 38.1, 12.7 | 9.7,  5.1 |  |  |  | 8 | Age; IQ |
| 20 | Bürk, 2006 | Germany | CSS | 20 MSA-C | D |  | 60.1, 5.0, range: 52-69 |  | 55.5, 5.0, range: 48-65 | 4.6, 2.6, range:  1-9 |  |  |  | 20 | Age; IQ |
| 21 | Chang, 2009 | Taiwan | CSS | 10 MSA-C | D | 5  (50%) | 57.1,  9.9 | 9.0,  5.9 y |  | 2.4 |  |  |  | 37 | Age; Sex; Education |
| 22 | Chirino, 2018 | Mexico | CSS | 31 SCA7 | D | 19 (61%) | 40.94, 14.09 | 7.05, 4.11 y | 34.00, 13.13 | 7.19, 4.85 | 14.66, 6.26 |  |  | 32 | Age; Sex; Education |
| 23 | Chirino-Pérez, 2021 | Mexico | CSS | 17 SCA10 | D | 9  (53%) | 49.23, 9.18 | 10.23, 2.58 y | 30.17, 7.07 | 19.00, 8.85 | 17.44, 6.24 |  |  | 17 | Age; Sex; Education |
| 24 | Clausi, 2021 | Italy | Experimental study | 36 degenerative cerebellar disease | D | 15 (42%) | 46.97, 10.17, range: 24-64 | 13.22, 3.2 y |  |  |  | 26.52, 12.4 |  | 67 | Age; Education |
| 25 | Cocozza, 2018 | Italy | CSS | 24 FA | D | 15 (63%) | 31.3, 15.0 | 12.1, 2.9 y |  |  | 18.7, 7.2 |  |  | 24 | Age; Sex; Education |
| 26 | Cook, 2004 | Australia | CSS | 5 left-sided cerebellar vascular lesions | F | 4  (80%) | 56.2, 12.9 | 8.8,  3.27 y |  | 1.82, 1.02 |  |  | 5  (100%) | 16 |  |
| 27 | Cooper, 2010 | UK | CSS | 27 SCA6 | D | 10 (37%) | 65, 9, range: 47-79 | 11,  2 y | 50,  22 | 9,  2 |  |  |  |  | Age |
| 28 | Corben, 2017 | Australia | CSS | 43 FA | D | 24 (56%) | 36, 12.2, range: 18-63 | 13.7, 2.2, range: 11-20 y | 18.4, 7.7, range: 6-40 | 17.4, 10, range:  5-48 |  |  |  | 42 | Age; Sex |
| 29 | Costabile, 2018 | Italy | CSS | 20 FA | D | 12 (60%) | 31.5, 14.7 | 12.4,  3.0 y |  | 16.7,  8.8 | 18.3, 6.1 |  |  | 20 | Age; Sex; Education |
| 30 | D'Agata, 2011 | Italy | CSS | 12 SCA2 | D | 10 (83%) | 48,  10 | 9,  3 y | 37,  10 | 11,  5 |  | 29,  5 | 12 (100%) | 31 | Age; Handedness |
| 31 | Daum, 1993 | Germany | CSS | 13 lesions restricted to cerebellum | Both | 8  (62%) | 53.4, 14.8 |  |  | 5.1,  4.5 |  |  | 13 (100%) | 13 | Age; IQ; mood scores; Handedness |
| 32 | de Nóbrega, 2007 | Spain | CSS | 24 FA | D | 12 (50%) | 40.54, 9.96 | 11.38, 4.11 y | 18.96, 11.01, range: 2-40 | 21.54, 10.50, range:  2-43 |  |  | 24 (100%) | 20 | Age; Education; Handedness |
| 33 | Dimitrov, 1996 | USA | Experimental study | 11 cerebellar atrophy | D |  | 55,  11 | 14,  2 y |  |  |  |  |  | 11 | Age; Education |
| 34 | Dirnberger, 2010 | Austria | CSS | 11 isolated cerebellar infarction | F | 5 (45.5%) | 46,  15 |  |  | 2.6, 1.5, range: 0.6-5.5 |  | 6,  4 |  | 13 | Age; Sex; MMSE; Handedness |
| 35 | Dogan, 2016 | Germany | CSS | 22 FA | D | 10 (45%) | 41.36, 13.57 | 3.59, 1.01 level (ISCED) | 20.95, 9.64 | 20.41, 11.04 | 18.61, 8.64 |  | 19  (86%) | 22 | Age; Sex; Education; Handedness |
| 36 | Erdal, 2021 | Turkey | Cohort study | 23 isolated cerebellar infarction | F | 17 (73.9%) | 52.2, 7.0 | 19 ≤8y,  4 >8y |  | 8.3,  0.9 |  |  | 21 (91.3%) | 22 | Age; Sex; Education; Handedness |
| 37 | Exner, 2004 | Germany | CSS | 11 cerebellar infarction | F | 6 (54.5%) | 58.8, 5.4 | 12.5, 2.4 y |  | > 6 m |  |  |  | 11 | Age; Sex; Education |
| 38 | Fan, 2019 | China | CSS | 40 cerebellar infarction | F | 31 (77.5%) | 61.43, 9.60 | 8.41, 2.21 y |  | < 2 m |  | 6.45, 2.33 |  | 40 | Age; Sex; Education; MMSE |
| 39 | Fancellu, 2013 | Italy | Cohort study | 42 SCA1 & SCA2 | D | 19 (45%) | 45.4, 10.0 | 9.6,  3.5 y |  | 9.8,  6.0 | 13.5, 6.5 |  | 40  (95%) | 17 | Age; Sex; Education |
| 40 | Fehrenbach, 1984 | Germany | CSS | 15 FA | D | 12 (80%) | 42.3, 11.6 |  |  | 28.4, 10.8 |  |  |  | 15 | Age; Sex; Education |
| 41 | Feng, 2014 | China | CSS | 22 SCA3 | D | 8  (36%) | 37.05, 6.10, range: 29-54 | 12.14, 3.21, range:  9-16 y | 32,  4.49, range: 25-40 | 5.15, 3.62, range:  1-14 |  | 28.14, 9.40 | 22 (100%) | 18 | Age; Sex; Education; Mood scores |
| 42 | Frank, 2010 | Germany | CSS | 22 unilateral cerebellar stroke | F | 20 (91%) | 52.9, 15.2, range: 24-77 |  |  | 0.037, 0.040, range:  1-57 d |  | 12.0, 9.0 | 21  (95%) | 22 | Age; Education; Handedness |
| 43 | Frommann, 2012 | Germany | Experimental study | 10 MSA-C | D | 6  (60%) | 61.50, 6.55 | 13.50, 4.06 y |  | 0.5, 0.7 |  |  |  | 26 | Age; Sex; IQ |
| 44 | Gama, 2019 | Brazil | CSS | 6 autosomal recessive ataxia (SYNE 1) | D | 3  (50%) | 43.3, 11 | 7.5,  4.6 y | 29.6 | 12.6 | 12.91, 9.69 | 30.83, 16.1 |  | 18 | Age; Education |
| 45 | Gambardella, 1998 | Italy | Cohort study | 6 SCA2 | D | 5 (83.3%) | 44.7, 15.9 | 6.7,  4.3 y | 42.5, 9.1 |  |  |  |  | 20 | Age; Sex; Education |
| 46 | García, 2022 | Spain | CSS | 38 hereditary ataxia | D | 22 (58%) | 50.37, 15.68 | 11.34, 2.49 y | 38.21, 16.20 | 23.71, 14.98 |  | 36.45, 21.66 |  | 38 | Age; Sex; Education |
| 47 | Geva, 2021 | UK | CSS | 4 focal cerebellar lesions | F | 2  (50%) | 44.5, 10.2 | 15.8,  2.6 y | 40.4, 11.1 | 4.13, 3.68 |  |  | 4  (100%) |  |  |
| 48 | Gigante, 2020 | Italy | CSS | 20 SCA2 | D | 9  (45%) | 53.4, 13.3 | 9.5,  3.4 y | 39.7, 13.6, range: 10-62 | 13.8, 10.6, range:  1-37 | 10.4, 4.5 |  | 20 (100%) | 20 | Age; Sex; Education |
| 49 | Globas, 2003 | Germany | CSS | 12 SCA6 | D | 5  (42%) | 57.5, 10.4, range: 39-70 |  | 47.9, 12.0, range: 25-65 | 9.2,  11.6, range:  2-42 |  |  |  | 12 | Age; Sex; IQ |
| 50 | Gottwald, 2004 | Germany | CSS | 21 cerebellar lesions due to tumour or haematoma | F | 11 (52.4%) | 52.6, 12.2, range: 26-71 | 12.5,  4.2 y |  |  |  |  | 18 (85.7%) | 21 | Age; Sex; Education |
| 51 | Harrison, 2019 | USA | CSS | 27 medulloblastoma | F | 18 (67%) | 33.1, 10.7, range: 20.6-63.4 | 14,  3.5 y, range: 6-20 | 32.5, 10.58, range: 20.5-62.8 | 0.345, range:  4-2031 d |  |  | 23  (85%) |  | Age; Education |
| 52 | Hart, 1985 | USA | CSS | 3 FA | D | 1  (33%) | 27, 9.2 | 12,  0 y |  |  |  |  |  | 6 | Age; Sex; |
| 53 | Hirono, 1991 | Japan | CSS | 30 spinocerebellar degeneration | D | 16 (53%) | 49, 8.8 | 11.8,  3.0 y |  | 7.2,  5.3 |  |  |  | 41 | Age; Sex; Education |
| 54 | Hoche, 2018 | USA | Diagnostic test accuracy study | 77 isolated cerebellar disease/injury & complex cerebrocerebellar disease | Both | 42 (54.5%) | 51.42, 14.1, range: 17-80 | 15.01, 1.35 y |  |  |  |  |  | 58 | Age; Sex; Education |
| 55 | Hokkanen, 2006 | Finland | Cohort study | 26 cerebellar infarction | F | 21 (80.8%) | 47,  11 | 13,  4 y |  | 0.019, 0.005, range:  4-11 d |  |  |  | 14 | Age; Education |
| 56 | Hong, 2011 | Korea | CSS | 26 MSA-C | D | 20 (77%) | 57.3,  7.5 | 12.1,  4.4 y | 54.1, 7.9 | 34.4, 15.7 |  |  |  | 26 | Age; Education |
| 57 | Jiang, 2013 | China | CSS | 10 focal cerebellar lesions (infarction, hemorrhage, or benign tumor) | F | 5  (50%) | 53.70, 13.94, range: 29-75 | 6.10, 3.90, range:  2-14 y |  | > 30 d |  |  |  | 10 | Age; Education |
| 58 | Jodzio, 2020 | Poland | CSS | 14 unilateral cerebellar infarctions | F | 11 (78.6%) | 53.93, 10.63 | 12.57, 3.18 y |  | > 3 d and < 30 d |  |  | 14 (100%) | 28 | Age; Sex; Education |
| 59 | Kansal, 2017 | USA | CSS | 72 cerebellar disease | D | 31 (43%) | 53.42, 13.40 | 15.90, 2.92 y |  |  |  |  |  | 36 | Age; Sex; Education |
| 60 | Karaci, 2008 | Turkey | CSS | 20 cerebellar vascular disease | F | 13 (65%) | 60.55, 10.17 |  |  | < 3 d |  |  |  | 20 | Age |
| 61 | Kawai, 2004 | Japan | CSS | 16 SCA3 | D | 7  (44%) | 48.2, 15.4, range: 28-73 | 12.3,  2.5, range: 8-16 y | 38.5, 13.5, range: 18-61 | 9.8,  4.5, range:  2-16 |  |  |  | 20 | Age; Sex; Education |
| 62 | Kawai, 2008 | Japan | CSS | 21 MSA-C | D | 12 (57%) | 60.3,  8.3, range: 48-74 | 12.1,  2.2, range: 9-17 y | 58.1,  8.8, range: 45-73 | 2.6,  1.6, range:  1-7 |  | 27.7, 11.8 |  | 21 | Age; Education |
| 63 | Kish, 1988 | Canada | CSS | 11 OPCA | D | 6  (55%) | 33,  8 | 13,  2 y |  | 10.7,  5.6 |  |  |  | 13 | Age; Sex; Education |
| 64 | Kish, 1994 | Canada | CSS | 43 spinocerebellar ataxia | D | 26 (60%) | 46,  12 | 13,  2 y |  | 11,  7 |  |  |  | 24 | Age; Sex; Education |
| 65 | Klinke, 2010 | Germany | CSS | 32 SCA 1, 2, 3 & 6 | D | 18 (56%) | 48.3, 11.6 |  | 41.2, 10.6 | 7.4,  4.7 | 9.7, 6.0 |  | 27  (84%) | 14 | Age; Sex; Education; IQ |
| 66 | Krygier, 2017 | Poland | CSS | 4 ARSACS | D | 1  (25%) | 38.8,  8.5 |  | 3.1,  1.7 | 35.5,  9.1 | 18.6, 3.9 |  |  |  |  |
| 67 | Laforce, 2010 | Canada | CSS | 21 ARCA-1 | D | 10 (48%) | 43.6, 8.3 | 12.5,  2.4 y | 31.5,  7.2 | 12.3,  7.3 |  |  |  | 21 | Age; Sex; Education |
| 68 | Le Pira, 2002 | Italy | CSS | 18 SCA2 | D | 9  (50%) | 48.06,  12.18, range: 26-67 | 6.44, 2.81, range: 4-13 y | 36.83, 19.64 | 11.22, 6.62 |  |  | 17  (94%) | 14 | Age |
| 69 | Lee, 2016 | Korea | CSS | 18 MSA-C | D | 11 (61%) | 58.9, 8.9, range: 48-80 | 9.3,  3.5 y |  | 2.53, 1.66 |  |  |  | 50 | Age; Education |
| 70 | Lilja, 2005 | Finland | CSS | 10 SCA8 | D | 2  (20%) | 39.9, 5.4, range: 32-48 | 13.4, 2.7, range: 10-17 y | 28.3,  4.4 |  |  |  |  | 10 | Age; Sex; Education |
| 71 | Lopes, 2013 | Brazil | CSS | 32 SCA3 | D | 15 (47%) | 46.78, 11.47 | 10.19, 3.78 y | 36.72, 10.91, range: 15-58 | 10.09, 5.78, range:  2-30 | 13.6, 6.3 |  |  | 32 | Age; Sex; Education |
| 72 | Ma, 2014 | China | CSS | 18 SCA 1, 2 & 3 | D | 12 (67%) | 45.67, 9.07 | 9.1,  4.3 y | 37.9,  7.3 | 8.7,  4.2 |  | 36.2, 19.5 |  | 16 | Age; Sex; Education; Nationality |
| 73 | Maddox, 2005 | USA | Experimental study | 6 unilateral focal lesions | F |  | 59.67, 10.89 | 13.83, 2.86 y |  |  |  |  |  | 14 | Age; Education |
|  |  |  |  | 8 degenerative cerebellar disease | D |  | 57.00, 13.18 | 16.25, 3.15 y |  |  |  |  |  | 14 | Age; Education |
| 74 | Mak, 2016 | Poland | CSS | 30 cerebellar cancer without metastases | F | 12 (40%) | 44.33, 11.89 | 12.66, 2.60 y |  | > 6 m |  |  |  | 30 | Age; Sex; Education |
| 75 | Martínez-Regueiro, 2020 | Spain | CSS | 19 SCA36 | D | 9  (47%) | 63,  11 | 8,  3 y |  | 12,  9 | 16.7, 10.1 |  | (100%) |  | Age; Sex; Education |
| 76 | Maruff, 1996 | Australia | Cohort study | 6 SCA3 | D | 4  (67%) | 41.8, range: 23-57 | 3,  range:  2-5 y |  |  |  |  |  | 15 | Age; Education; Ethnicity |
| 77 | Maschke, 2002 | Germany | Experimental study | 8 degenerative cerebellar disorder | D | 6  (75%) | 52.2,  6.2 |  |  | 15.4,  9.5 |  |  | 7  (88%) | 8 | Age; Sex; Education; IQ |
| 78 | Mastammanavar, 2020 | India | Prospective case-control study | 41 SCA 1, 2 & 3 | D | 27 (66%) | 32.9,  9.2 |  | 28.3,  8.4 | 4.5,  3.5 |  | 36.3, 16.9 |  | 48 | Age; Sex |
| 79 | Meles, 2018 | The Netherlands | CSS | 17 SCA3 | D | 9  (54%) | 45.3, 11.4, range: 24-62 | 5, 0.25 level (Verhage) | 35.6, 10.2, range: 20-55 | 9.7,  7,  range:  3-30 | 10,  3 |  |  | 16 | Age; Sex; Education |
| 80 | Molinari, 2004 | Italy | CSS | 25 cerebellar lesions | F |  | 51.38, 16.85 | 9.79, 4.34 y |  |  |  |  | 25 (100%) | 21 | Age; Education |
|  |  |  |  | 14 degenerative disease | D |  | 39.00, 13.94 | 9.14, 3.11 y |  |  |  |  | 14 (100%) | 10 | Age; Education |
| 81 | Moro, 2016 | Brazil | Cohort study | 28 SCA10 | D | 13 (46%) | 46.8, 11.6 | 11.1,  4.1 y | 31.7,  7.6 | 15.5,  12 | 9.9, 4.5 |  |  | 28 | Age; Sex; Education |
| 82 | Nachbauer, 2014 | Austria | CSS | 29 FA | D | 18 (62%) | 34.48, 12.34 | 12.21, 2.27 y | 17.76, 10.08 | 16.69, 8.92 | 20.47, 7.89 |  | 27  (93%) | 28 | Age; Sex; Education; IQ |
| 83 | Neau, 2000 | France | CSS | 15 isolated cerebellar infarcts | F | 9  (60%) | 57.1, 12.8, range: 39-75 | 3.9,  1.7, range:  2-7 level |  | 0.044, 0.047, range:  3-75 d |  |  | 15 (100%) | 15 | Age; Sex; Education |
| 84 | Nieto, 2012 | Spain | CSS | 36 FA | D | 20 (56%) | 33.94, 12.23 | 12.39, 4.09 y | 18.06, 9.40 | 15.89, 8.63 |  |  | 32  (89%) | 31 | Age; Sex; Education; IQ; MMSE |
| 85 | Orsi, 2011 | Italy | CSS | 33 spinocerebellar ataxia | D | 18 (54%) | 49,  10 | 8,  4 y | 37,  10 | 13,  12 |  | 38,  14 |  | 20 | Age; Sex; Education |
| 86 | Peterburs, 2010 | Germany | CSS | 14 focal vascular cerebellar lesions | F | 8  (57%) | 54.6, 17.2, range: 25-74 |  | 52.8, 17.8, range: 24-73 | 1.675, 2.942, range:  3-141 m |  |  |  | 14 | Age; Sex; IQ |
| 87 | Rentiya, 2018 | USA | CSS | 30 SCA6 & ILOCA | D | 8  (27%) | 57.93, 9.44 | 15.45, 3.17 y |  | 10.64, 9.70 |  | 32.36, 16.62 |  | 27 | Age; Sex; Education |
| 88 | Reumers, 2024 | The Netherlands | Cohort study | 58 cerebellar stroke | F | 31 (52%) | 41.64, 6.88 | 5.47, 1.08 level (Verhage) | 41.35, 6.89 | 104.29, 58.73 |  |  |  |  | Age; Sex; Education |
| 89 | Richter, 2004 | Germany | Experimental study | 12 cerebellar disease | D | 10 (83%) | 50.3, 10.4, range: 40-75 |  |  | 14.1,  7.5 |  | 36, 20.1 | 11  (92%) | 12 | Age; Sex; Education; Handedness |
| 90 | Richter, 2007 | Germany | CSS | 21 focal cerebellar lesions | F | 16 (76%) | 55.1, 11.1, range: 34-71 |  |  | 3.89, 1.49, range: 17-96 m |  | 3.38, 5.77 | 17  (81%) | 25 | Age; Sex |
| 91 | Rodríguez-Labrada, 2014 | Germany | Experimental study | 41 SCA2 | D | 21 (51%) | 39.32, 10.15, range: 15-63 |  | 28.13, 9.19, range:  9-55 | 11.52, 5.96, range:  4-26 | 16.40, 3.55 |  |  | 41 | Age; Sex |
| 92 | Santangelo, 2020 | Italy | Cohort study | 21 MSA-C | D | 11 (52%) | 60.8,  7.8 | 9.9,  4.6 y |  | 4.8,  3 |  |  |  | 30 | Age; Sex; Education; MoCA |
| 93 | Satoer, 2024 | The Netherlands | CSS | 43 cerebellar stroke | F | 18 (41.9%) | 62.65, 15.53, range: 22-92 | 12.41, 3.46, range:  6-22 y |  | 3 m |  |  | 35 (81.4%) |  | Age; Sex; Education |
| 94 | Sayah, 2018 | France | Cohort study | 47 FA | D | 18 (38%) | 37.9, 13.4, range: 18-76 | 13.3,  2.2, range:  8-17 y | 20.4, 12.9, range:  3-65 | 16.5,  8.2, range:  4-36 | 22.9, 9.4 |  |  |  | Age; Sex; Education |
| 95 | Schmahmann, 1998 | USA | CSS | 20 diseases confined to cerebellum | Both | 12 (60%) | 48.2, range: 23-74 | 13.9 y |  | Range:  1 w - 6 y |  |  |  |  | Age; Sex; Education |
| 96 | Schweizer, 2007 | Canada | Experimental study | 11 focal cerebellar lesions | F | 7  (64%) | 48.3, 16.95 range: 24-74 | 13.18, 4.00 y |  | 1.238, range: 90-980 d |  |  |  | 13 | Age; Sex; Education |
| 97 | Shen, 2022 | China | CSS | 57 MSA-C | D | 40 (70.2%) | 55.81, 8.10 | 10.90, 3.14 y | 53.85, 8.19 | 1.96, 1.44 |  |  |  |  | Age; Education |
| 98 | Shin, 2017 | Korea | CSS | 26 cerebellar stroke | F | 17 (65%) | 54.8, 16.6 |  |  | 0.73, 0.77 |  |  | 26 (100%) |  | Age; Education |
| 99 | Shin, 2024 | South Korea | Cohort study | 29 degenerative cerebellar ataxia | D | 13 (44.8%) | 59.34, 8.33 | 11.28, 3.46 y |  | 8.10, 5.99 | 12.37, 5.80 |  |  | 23 | Age; Sex; Education |
| 100 | Shishegar, 2020 | Australia | Cohort study | 21 FA | D | 15 (71%) | 35.23, 2.65 | 14.38, 0.41 y | 21.24, 2.06 | 13.83, 1.49 |  |  |  | 28 | Age; Sex |
| 101 | Siciliano, 2022 | Italy | CSS | 12 SCA2 | D | 5  (42%) | 48.3,  8.3 | 14.4, 3.7 y |  | 7.09, 4.48 |  | 31.75, 11.91 |  |  | Age; Education |
| 102 | Slapik, 2019 | USA | CSS | 49 cerebellar ataxia | D | 21 (43%) | 56.22, 11.08 | 16.38, 2.78 y |  |  |  | 33.86, 14.26 (n=32) | 37  (95%) (n=39) | 60 | Age; Sex; Education |
| 103 | Starowicz-Filip, 2021 | Poland | CSS | 28 cerebellar lesion | F | 11 (39%) | 51.76, 17.39 |  |  | 0.345, 0.368 |  |  |  | 31 | Age; Sex; Education |
| 104 | Stoodley, 2009 | USA | CSS | 18 cerebellar degeneration | D | 10 (56%) | 45.8, 13.7 | 14.8,  1.9 y |  |  |  | 40.5, 21.8 |  | 16 | Age; Education; IQ |
| 105 | Stoodley, 2016 | USA | CSS | 18 isolated cerebellar stroke | F | 13 (72%) | 46.8, 14.6, range: 20-66 |  | 46.8, 14.6, range: 20-66 | 0.082, 0.050, range:  8-67 d |  | 9.1, 8.8 |  |  | Age |
| 106 | Storey, 1999 | Australia | Cohort study | 8 SCA2 | D | 4  (50%) |  |  | 33.5, 11.5, range: 21-46 | 9.3,  5.2, range:  4-20 |  |  |  |  |  |
| 107 | Suenaga, 2008 | Japan | CSS | 18 SCA6 | D | 9  (50%) | 64.1, 11.9, range: 37-81 | 11.1,2.3, range: 9-16 y | 52.1, 11.7, range: 30-75 | 12.6,  8.2, range:  3-30 |  | 36.4, 12.0 |  | 21 | Age; Education |
| 108 | Szpisjak, 2017 | Hungary | Cohort study | 5 SCA28 | D | 2  (40%) | 41.60, 16.43 | 18.2, 4.44 y | 18.2, 5.50 |  | 8.70, 3.55 |  |  |  | Age |
| 109 | Tamaš, 2021 | Serbia | CSS | 34 cerebellar neurodegenerative ataxia | D | 20 (59%) | 48.9, 11.8 | 12.7,  2.1 y | 39.7, 12.3 | 9.1,  7.4 | 12.3, 4.9 |  |  | 34 | Age; Sex; Education |
| 110 | Tamura, 2017 | Japan | CSS | 13 SCA6 | D | 9  (69%) | 65.3, 9.92, range: 48-80 | 11.46, 1.85, range:  8-14 y | 52.39, 10.3, range: 37-70 | 14.25, 5.96, range:  4-24 | 16.5, 8.2 |  | 13 (100%) | 13 | Age; Education; MMSE |
| 111 | Tamura, 2018 | Japan | CSS | 15 SCA3 | D | 9  (60%) | 53.80, 11.79, range: 35-72 | 12.27, 2.49, range:  9-19 y | 42.3, 12.2, range: 26-61 | 11.5,  7.8, range:  2-31 | 17.4, 8.0 |  | 15 (100%) | 15 | Age; Sex; Education; IQ |
| 112 | Tanaka, 2003 | Japan | Experimental study | 13 cortical cerebellar atrophy | D | 7  (54%) | 59.1, 10.0 | 11,  2.3 y |  | 8.4, 7.6 |  |  |  | 13 | Age; Sex; Education |
| 113 | Thomasson, 2019 | Switzerland | CSS | 15 ischaemic cerebellar stroke | F | 9  (60%) | 63.5,  9.6, range: 50-77 | 16.4,  4.7, range:  9-22 y |  | > 3 m | 2.4, 3.0  (n=11) |  | 13  (87%) | 15 | Age; Sex; Education; Handedness |
| 114 | Timmann, 2004 | Germany | Experimental study | 8 degenerative cerebellar disease | D | 6  (75%) | 52.15, 6.16, range: 39-66 |  |  | 15.4,  9.5 |  |  | 7  (88%) | 8 | Age; Sex; Education; IQ |
| 115 | Torrens, 2008 | UK | Cohort study | 10 SCA8 | D | 4  (40%) | 51.8, 12.2 |  | 42.2, 12.3 |  |  |  |  | 10 | Age; Sex; IQ |
| 116 | Turner, 2007 | USA | Experimental study | 6 cerebellar stroke | F | 6 (100%) | 67.3, 4.0 | 13.7,  3.3 y |  | Range:  14-180 d |  |  |  | 9 | Age; Education ; IQ |
| 117 | Vaca-Palomares, 2015 | Cuba | CSS | 41 SCA2 | D | 25 (63%) | 40.3,  9.9, range: 22-62 | 11.1,  3.1, range:  3-16 y | 29.9,  9.2, range: 15-55 | 10.7,  5.4, range:  2-26 |  |  | 41 (100%) | 34 | Age; Sex; Education; Handedness |
| 118 | Valis, 2011 | Czech Republic | CSS | 12 SCA2 | D | 7  (58%) | 49.8,  9.1, range: 35-62 | 12.1,  2.3, range:  8-18 y | 41.1,  9.7, range: 23-57 | 8.7,  5.7, range:  2-20 |  |  |  | 12 | Age; Sex |
| 119 | van den Berg, 2020 | The Netherlands | CSS | 13 isolated cerebellar stroke | F | 11 (85%) | 61.4, 10.9, range: 43-76 | 4.5, 1.1 level (Verhage scale) |  | Range:  1-40 m |  |  |  | 106 | Age; Sex; Education |
| 120 | van der Giessen, 2023 | The Netherlands | Cohort study | 52 isolated cerebellar lesions | F | 32 (63%) | 62.8, 12, range: 21-91 | 12.8,  4 y |  | 3 m |  | 5.9, 7.8 |  |  | Age; Sex; Education |
| 121 | Verbitsky, 2023 | Canada | CSS | 20 isolated cerebellar stroke | F | 15 (75%) | 55.51, 12.90, range: 31-79 |  |  | 45.40, 124.51, range:  1-566 |  |  | 18  (90%) |  | Age |
| 122 | Wallesch, 1990 | Germany | CSS | 12 cerebellar hemisphere lesions | F | 9  (75%) | 39.4, 15.6 |  |  | 2.875, 1.783, range:  7-61 m |  |  | 12 (100%) | 12 | Age; Sex; Education |
| 123 | Wang, 2022 | China | CSS | 37 cerebellar infarction | F | 21 (57%) | 61.24, 6.12 | 8.00, 1.40 y |  | Range:  4-14 d |  |  |  | 27 | Age; Sex; Education |
| 124 | White, 2000 | Canada | CSS | 15 FA | D | 8  (53%) | 36.1,  6.2 | 12.3,  3.1 y |  | 24.1, 18.2 |  |  | 12  (80%) | 15 | Age; Sex; Education |
| 125 | Witt, 2002 | Germany | Experimental study | 16 cerebellar degeneration | D | 10 (63%) | 53.38, 14.71 | 9.93,  1.0 y |  |  |  |  |  | 20 | Age; Sex; Education; IQ |
| 126 | Wollmann, 2002 | Spain | CSS | 12 FA | D | 4  (33%) | 29.58, 8.12 | 6.58, 2.15 y | 11.25, 5.8 | 17.5,  8.5 |  |  | 11  (92%) | 12 | Age; Sex; Education; Handedness |
| 127 | Yang, 2013 | USA | Experimental study | 41 FXTAS | D | 29 (71%) | 61.9,  8.1 | 15.6, 3.0 y |  |  |  |  | 36  (88%) | 32 | Age; Sex |
| 128 | Ye, 2023 | China | CSS | 126 SCA3 | D | 81 (64%) | 40.5,  8.3 | 12, 1.7 y (n=123) | 36,  7.8 | 6,  6.7 | 8,  4.5 | 20.5, 11.3 |  | 41 | Age; Sex; Education |
| 129 | Zawacki, 2002 | USA | CSS | 6 SCA3 | D | 3  (50%) | 56.5, 8.2, range: 43-64 | 13.3, 2.4 y | 45.3, 8.1 | 11.2 |  |  |  |  | Age; Sex; Education |

Continuous variables are presented as means with standard deviations, or numbers with percentages.
Empty cells reflect non-reported data.
CCS = Cross sectional study; SCA = Spinocerebellar Ataxia; OPCA = Olivopontocerebellar Atrophy; MSA-C = Multiple System Atrophy, Cerebellar type; FA = Friedreich's ataxia; FXTAS = Fragile X-Associated Tremor/Ataxia Syndrome; ARSACS = Autosomal Recessive Spastic Ataxia of Charlevoix-Saguenay; ARCA-1 = Autosomal Recessive Cerebellar Ataxia Type 1; ILOCA = Idiopathic Late Onset Cerebellar Ataxia, D = Degenerative cerebellar ataxia, F = Focal cerebellar lesions.
