# Supplementary material for: Cognition in cerebellar disorders: What’s in the profile? A systematic review and meta-analysis
Source: J Neurol. 2025 Mar 6;272(3):250. doi: 10.1007/s00415-025-12967-8 (PMC11885410; doi:10.1007/s00415-025-12967-8)
Supplement: Supplementary file 5 — Supplementary file5 (DOCX 3068 KB) [file 415_2025_12967_MOESM5_ESM.docx]

# **Forest and funnel plots per cognitive domain**


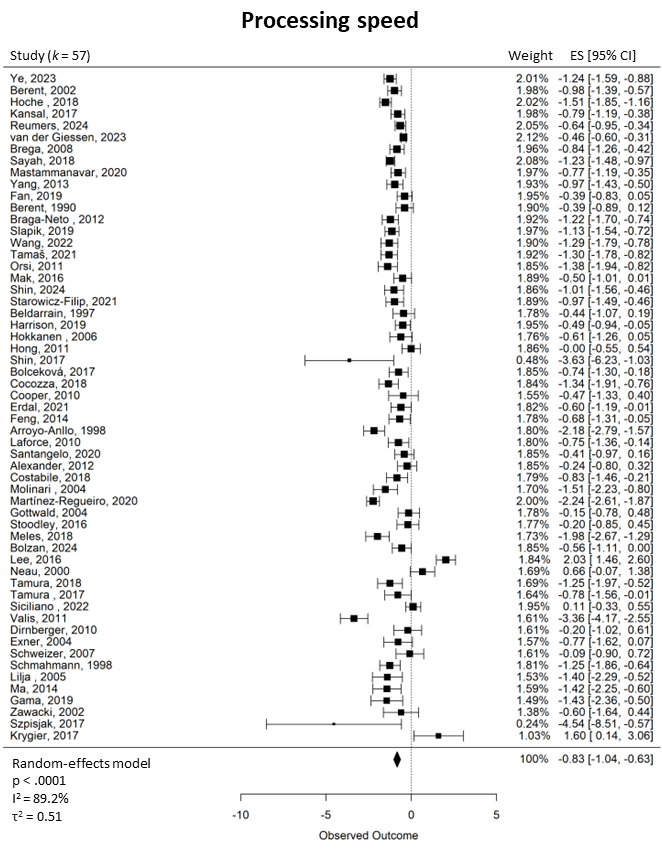


## **Processing speed**


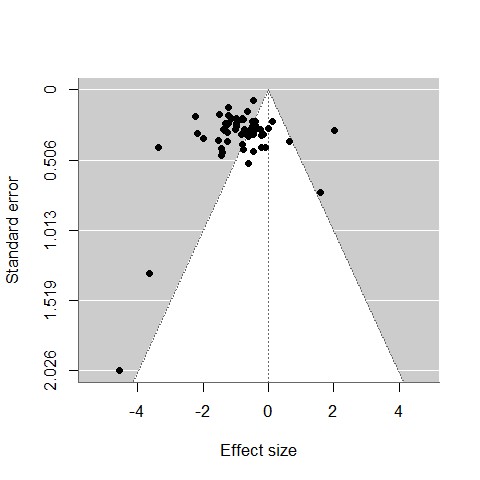


**
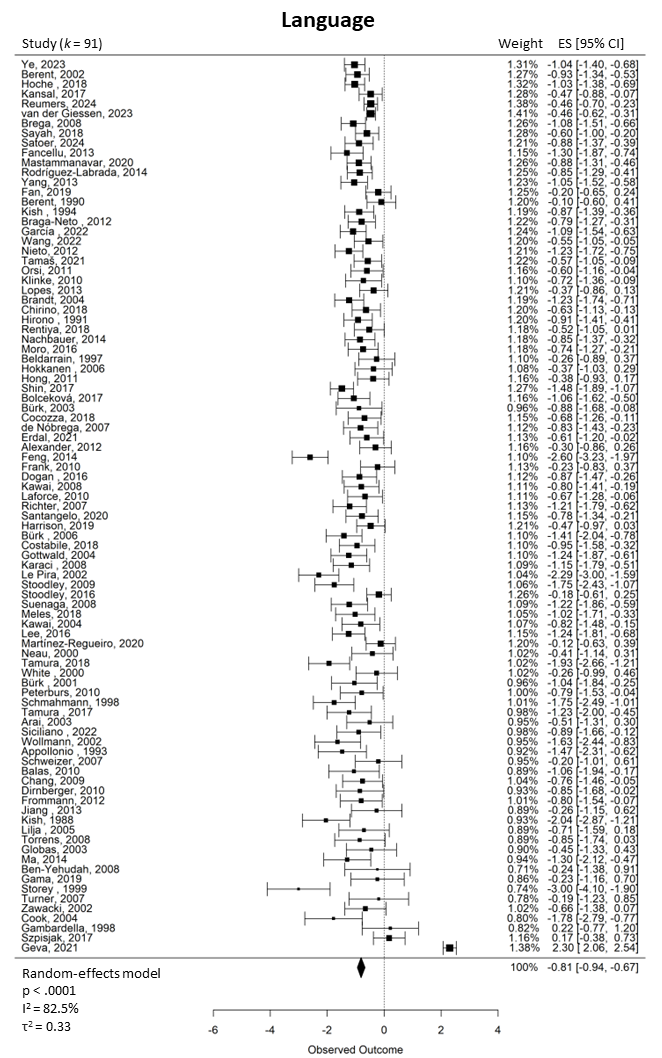
**

**Language**

**
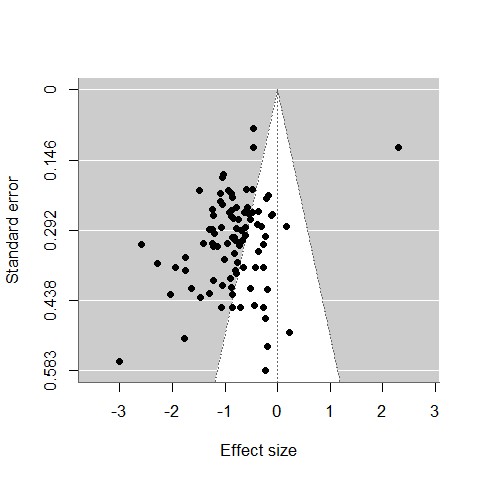
**

**
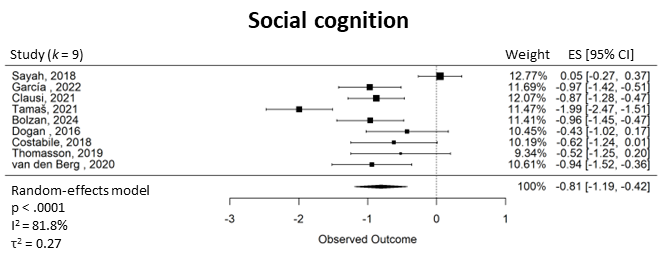
**

**Social cognition**

**
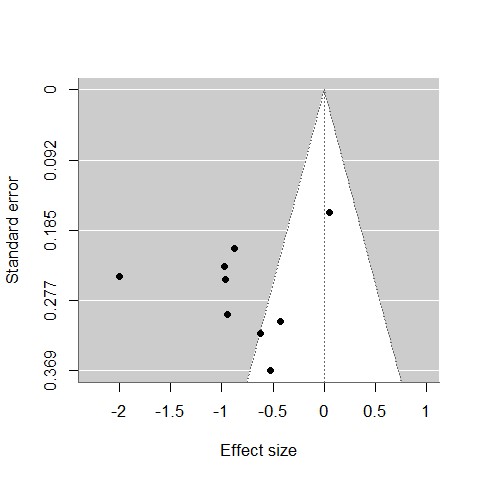
**

**
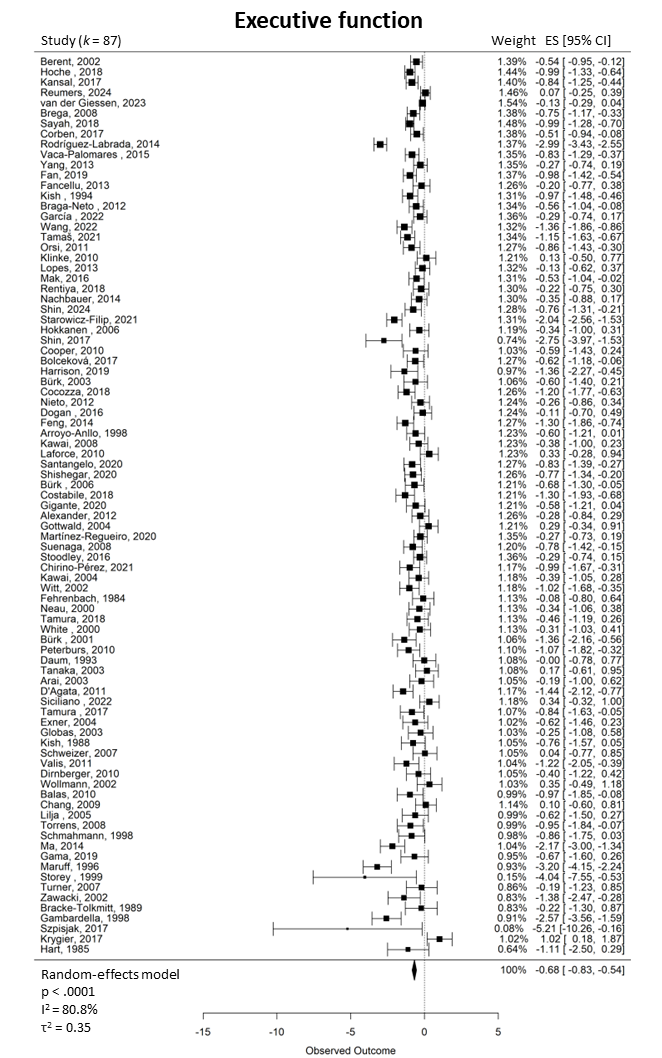
**

**Executive function**

**
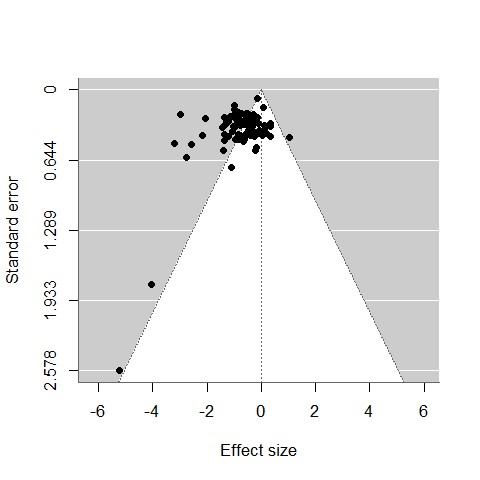
**

**
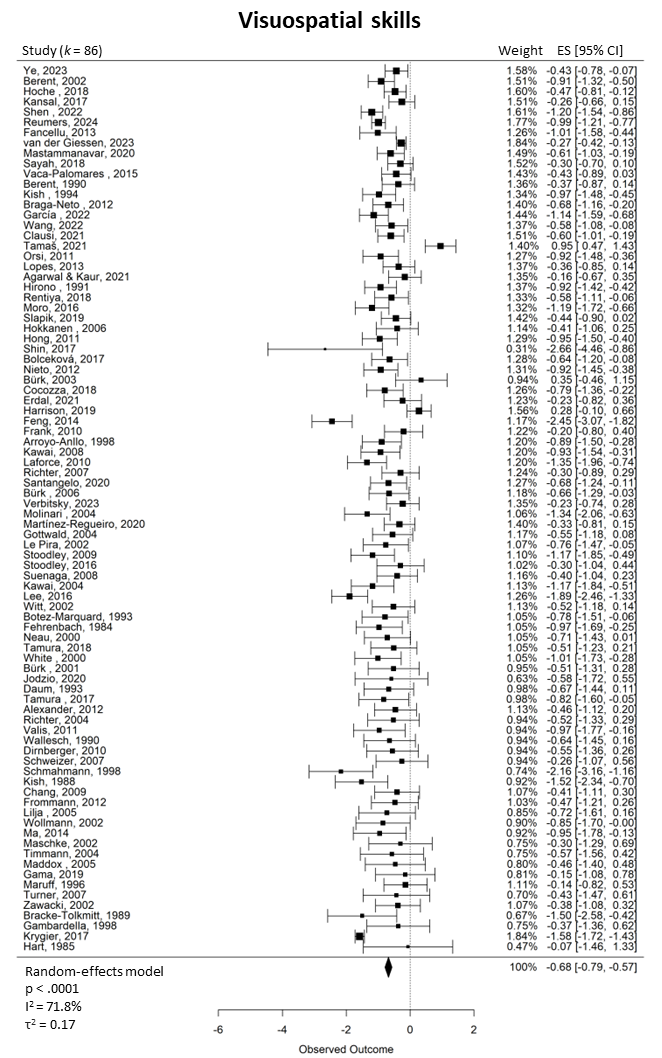
**

**Visuospatial skills**

**
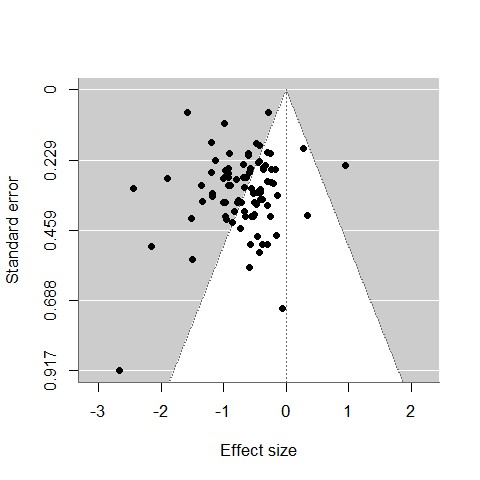
**

**
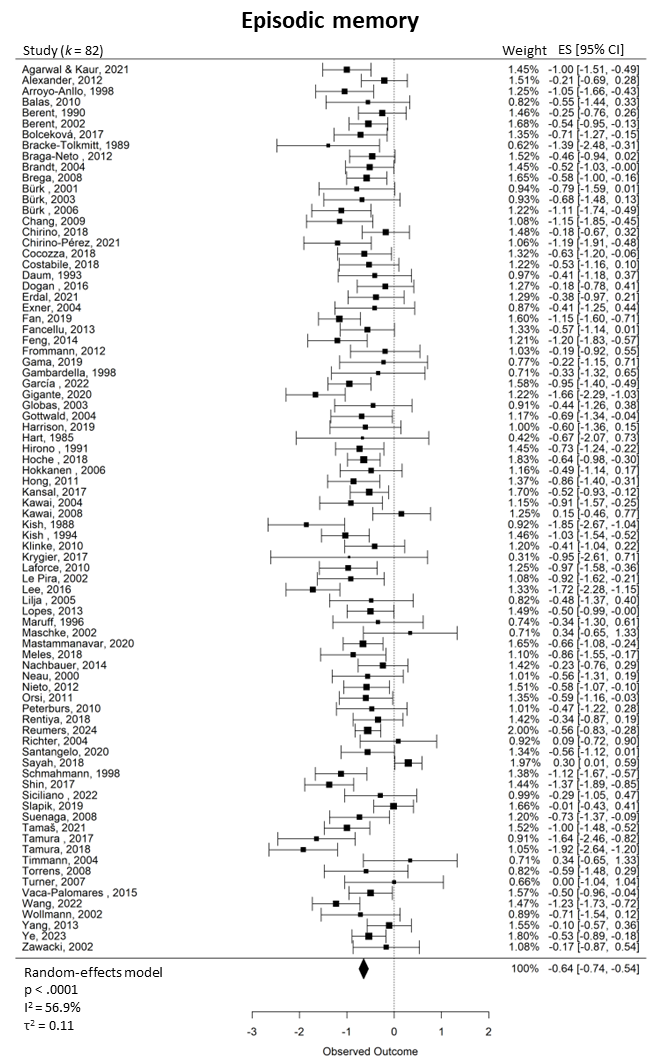
**

**Episodic memory**

**
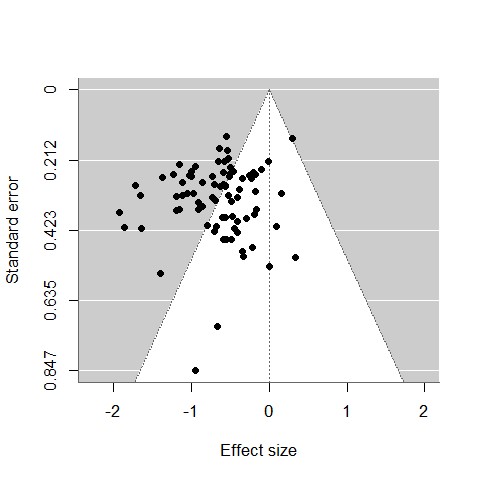
**

**
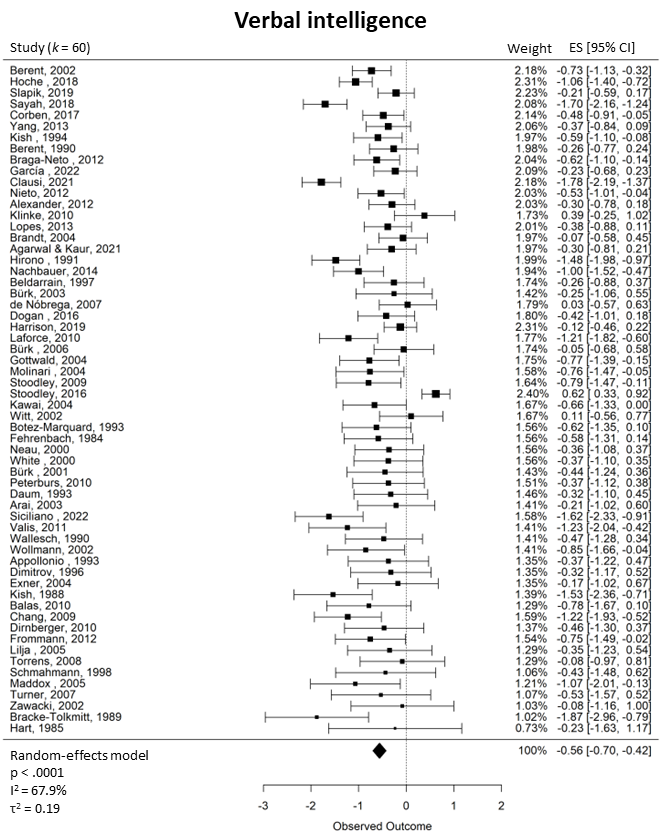
**

**Verbal intelligence**

**
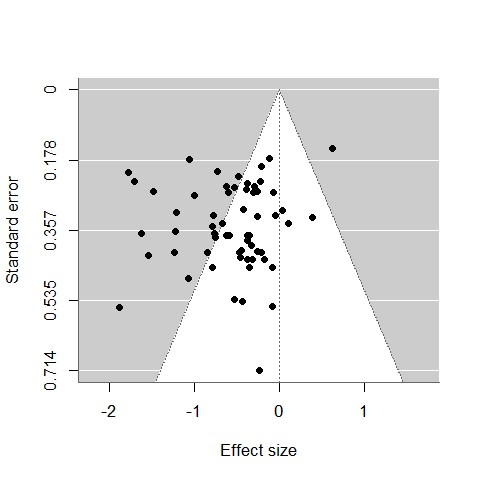
**

**
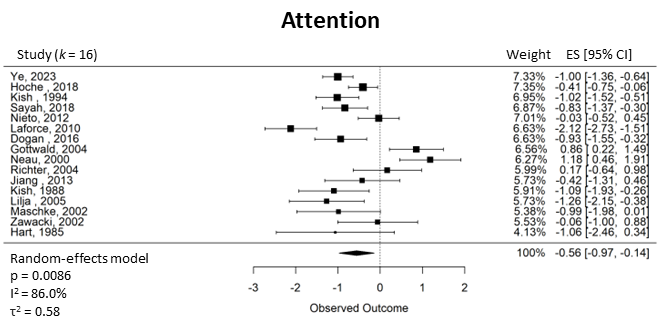
**

**Attention**

**
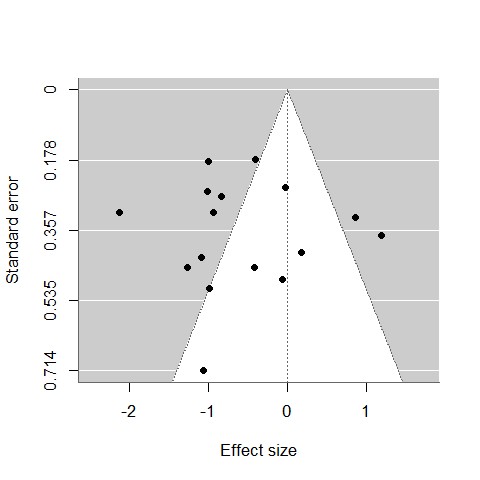
**

**
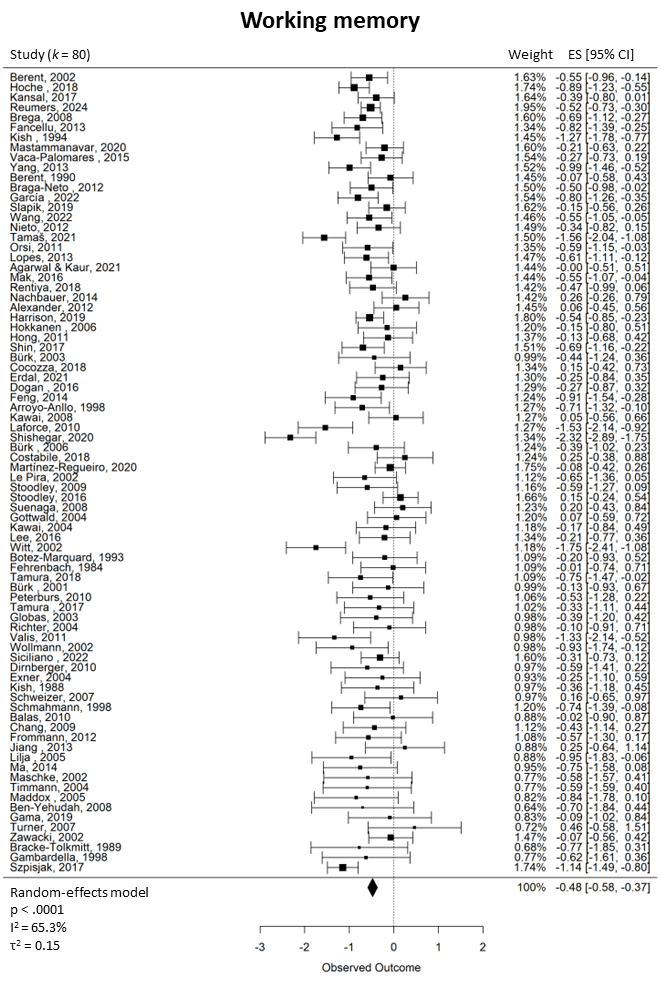
**

**Working memory**


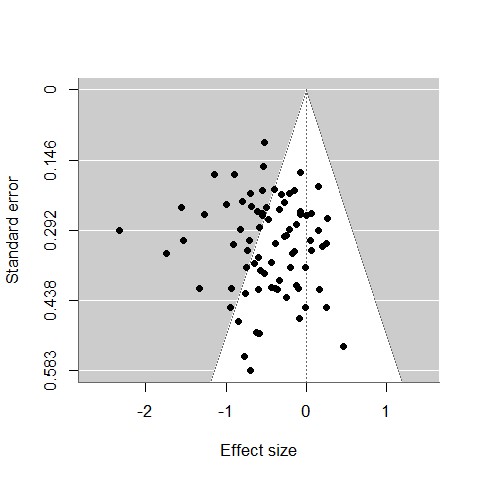


Squares represent the effect estimates of individual studies, with corresponding size to the weight. Horizontal lines represent the 95% confidence intervals. The diamond shape represents the pooled result for all studies combined.
ES = effect size
