# Supplementary material for: Cognition in cerebellar disorders: What’s in the profile? A systematic review and meta-analysis
Source: J Neurol. 2025 Mar 6;272(3):250. doi: 10.1007/s00415-025-12967-8 (PMC11885410; doi:10.1007/s00415-025-12967-8)
Supplement: Supplementary file 6 — Supplementary file6 (DOCX 16 KB) [file 415_2025_12967_MOESM6_ESM.docx]

# **Effect sizes with 95% CI per subdomain**

| Cognitive domain  Subdomain | ES | 95% CI | *p* | *k* |
| --- | --- | --- | --- | --- |
| Language  Word production  Naming  Comprehension  Reading | -0.91  -0.43  -0.27  -0.24 | -1.05, -0.76  -0.62, -0.25  -0.49, -0.06  -1.38, 0.91 | < .0001  < .0001  0.0126  0.6871 | 81  30  9  1 |
| Social cognition Theory of mind  Emotion recognition | -0.83  -0.49 | -0.42, -0.24  -0.90, -0.08 | 0.0056  0.0185 | 6  4 |
| Executive function  Shifting  Response inhibition Planning | -0.70  -0.53  -0.43 | -0.85, -0.55  -0.79, -0.27  -0.71, -0.15 | < .0001  < .0001  0.0026 | 72  41  11 |
| Visuospatial skills  Visuospatial reasoning/ construction  Perception  Visual scanning | -0.74  -0.42  -0.55 | -0.86, -0.62  -0.55, -0.29  -0.81, -0.30 | < .0001  < .0001  < .0001 | 78  19  9 |
| Episodic memory Immediate memory scale  Immediate verbal learning and memory  Immediate visuospatial learning and memory  Delayed memory scale  Delayed verbal learning and memory  Delayed visuospatial learning and memory | -0.69  -0.72  -0.58  -0.63  -0.58  -0.66 | -0.84, -0.55  -0.88, -0.56  -0.74, -0.42  -0.79, -0.46  -0.70, -0.54  -0.83, -0.48 | < .0001  < .0001  < .0001  < .0001  < .0001  < .0001 | 26  53  35  25  45  24 |
| Attention  Sustained attention  Alertness  Divided attention / dual-tasking | -0.81  -0.37  -0.34 | -1.35, -0.26  -1.50, 0.76  -1.17, 0.50 | 0.0038  0.5196  0.3370 | 7  2  6 |

The cognitive domains of processing speed, verbal intelligence, and working memory had no subdomains.
